# Digital Reminiscence for Predeath Grief Among Family Caregivers of Patients With Dementia: A Pilot Randomized Clinical Trial

**DOI:** 10.1001/jamanetworkopen.2026.8278

**Published:** 2026-04-22

**Authors:** Francesca B. Falzarano, Annabelle Greenfield, Sydney C. Saviano, Sindhu Kolla, Sosi Korian, Francesco Osso, Joseph Miller, Heather E. Whitson, Paul K. Maciejewski, Holly G. Prigerson

**Affiliations:** 1 Leonard Davis School of Gerontology, University of Southern California, Los Angeles; 2 Department of Radiology, Cornell Center for Research on End-of-Life Care, Weill Cornell Medicine, New York, New York; 3 Department of Psychology, Fordham University, Bronx, New York; 4 Duke Aging Center and Duke–University of North Carolina Alzheimer’s Disease Research Center, Duke University School of Medicine, Durham; 5 Geriatrics Research Education and Clinical Center, Durham Veterans Affairs Medical Center, Durham, North Carolina

## Abstract

**Importance:**

Family caregiving for individuals with Alzheimer disease and related dementias (ADRD) is associated with significant distress related to physical, emotional, and interpersonal losses. Reminiscence therapy may help family caregivers preserve connection, reframe loss, and reduce predeath grief, although its evaluation among caregivers remains underexplored.

**Objective:**

To assess the feasibility, acceptability, and preliminary efficacy of a digital reminiscence platform (Living Memory Home for Dementia Care Pairs [LMH-4-DCP]) for ADRD caregiver–care recipient dyads.

**Design, Setting, and Participants:**

This multisite, participant-blinded pilot randomized clinical trial was conducted virtually at Weill Cornell Medicine, New York, New York, and the University of Southern California, Los Angeles. Eligibility included being a primary caregiver for a family member with ADRD, age 18 years or older, and fluency in English. Participants were randomized in a 1:1 ratio and completed baseline and 2-week postintervention assessments. Enrollment occurred between November 6, 2024, and April 15, 2025. Data analyses were conducted from August 1 to 31, 2025.

**Intervention:**

LMH-4-DCP is a digital intervention for ADRD caregiver–care recipient dyads to engage in reminiscence activities. Participants were randomized to the LMH-4-DCP or an attention control condition, which included a restricted version of the platform without reminiscence features.

**Main Outcomes and Measures:**

Outcomes, including feasibility, acceptability, predeath grief, and relationship quality were assessed at baseline and 2-week follow-up.

**Results:**

Of 174 screened caregivers, 106 were eligible, and 68 enrolled. Among the enrolled participants, the mean (SD) age was 49.4 (13.7) years (range, 23-90 years). Fifty-four participants (79.4%) were female, and 39 (57.4%) were caring for a parent. Fifty-four randomized participants completed follow-up assessments (27 per group). Feasibility and acceptability of LMH-4-DCP was demonstrated by a study completion rate of 120 of 136 assigned assessments (88.2%). Most participants in the intervention group reported that LMH-4-DCP was easy to use (20 of 27 [74.1%]) and had well-integrated features (21 of 27 [77.8%]). Intervention participants showed reduced predeath grief (Cohen *d* = −0.38; mean [SD] reduction, −3.29 [8.64]; *P* = .03) after 2 weeks of using LMH-4-DCP. Compared with controls, intervention participants demonstrated greater reductions in predeath grief. Improvements in relationship quality were observed for intervention participants, but the difference was not significant.

**Conclusions and Relevance:**

This pilot randomized clinical trial demonstrated the feasibility, acceptability, and preliminary efficacy of LMH-4-DCP in reducing caregiver grief, with improvements in caregiver–care recipient relationship quality. These findings should inform platform refinements and a future, definitive efficacy trial.

**Trial Registration:**

ClinicalTrials.gov Identifier: NCT06225986

## Key Points

**Question:**

Is a digital reminiscence intervention for family caregivers of patients with dementia and their care recipients feasible, acceptable, and associated with changes in predeath grief and relationship quality?

**Findings:**

This pilot randomized clinical trial with 68 caregiver–care recipient dyads found that the Living Memory Home for Dementia Care Pairs (LMH-4-DCP) platform was a feasible and acceptable digital intervention that demonstrated preliminary efficacy for reducing caregiver predeath grief.

**Meaning:**

LMH-4-DCP was found to be a promising intervention for family caregivers to reduce predeath grief, supporting further evaluation in a confirmatory trial.

## Teaser

This pilot randomized clinical trial assesses the feasibility, acceptability, and preliminary efficacy of the Living Memory Home for Dementia Care Pairs platform as an intervention for family caregivers of patients with Alzheimer disease and related dementias.

## Introduction

Nearly 12 million family caregivers in the US assume primary responsibility for meeting the emotional, physical, and practical needs of 7 million Americans with Alzheimer disease or related dementias (ADRD).^1^ As ADRD progresses, caregivers suffer interpersonal losses, including emotional reciprocity, mutual recognition, and meaningful connection with the care recipient, in addition to anticipatory grief as they consider the patient’s future and eventual death.^2^ As formerly mutual relationships shift to predominantly unidirectional roles, caregivers experience relational deprivation and eroding relational bonds with the care recipient.^3^ These interpersonal losses contribute to predeath grief—a form of mourning, characterized by longing, yearning, and role and identity confusion that emerges prior to the care recipient’s death.^4,5^

Despite the care recipient’s physical presence, predeath grief and relational deprivations yield psychosocial disruptions analogous to those experienced in bereavement.^6^ Predeath grief negatively predicts caregiver outcomes (eg, depression, anxiety,^7,8,9^ burden,^10^ impaired medical decision-making^9,11^). Caregivers for patients with ADRD may experience predeath grief at a greater intensity than postloss grief,^6,12,13^ which increases risk for Prolonged Grief Disorder (PGD).^14^ However, predeath grief remains absent from prevailing models of stress and burden for caregivers of patients with dementia, despite intervention and supports being most needed before death in the context of ADRD caregiving.^6^

Reminiscence therapy (RT) facilitates prompt-based autobiographical memory recall.^15,16^ Evidence of its effectiveness remains mixed due to inconsistencies in delivery, setting, duration, and facilitation and has largely examined group-based RT within long-term care,^17^ while evaluation of caregiver outcomes remains limited. The microsociological theory of adjustment to loss highlights the importance of addressing psychosocial voids related to ADRD losses,^18^ while the interdependence model of communal coping (IMCC) posits that shared activities can strengthen relational bonds and reduce stress by fostering emotional connection and meaning making (ie, dyads can derive meaning from past and present moments together).^19,20^ Consistent with these theories, higher relationship quality is linked to better dyadic outcomes (reduced burden for caregivers, fewer neuropsychiatric symptoms for patients).^21,22^ By emphasizing positive relational experiences, RT may promote cognitive reframing, and thus, ameliorate predeath grief,^23^ underscoring its promise as a strengths-based, meaning-centered intervention to preserve connection and reduce caregiver distress.

The present study adapts the original digital postdeath bereavement platform (P.L.M. and H.G.P.; Living Memory Home [LMH]^24^; National Institute of Mental Health), comprised of reflective journaling and imagined dialogue activities into a predeath RT intervention for ADRD caregiver–care recipient dyads. Prior research on the LMH bereavement platform showed associations between reminiscence-focused journaling and reduced PGD severity from 1-week to 1-month follow-up.^25^ The present intervention (Living Memory Home for Dementia Care Pairs [LMH-4-DCP]) incorporates RT to promote collaborative engagement, assisting family caregivers in documenting the life story of those with mild to moderate ADRD to reduce predeath grief and improve the relationship of the caregiver and care recipient. Thus, the present study aims to test the feasibility, acceptability, and preliminary efficacy of the LMH-4-DCP intervention for family caregivers of patients with ADRD.

## Methods

### Study Design and Settings

Data collection for this multisite pilot randomized clinical trial (RCT) occurred virtually at Weill Cornell Medicine, New York, New York (primary study site), and the University of Southern California, Los Angeles (secondary study site). Participation lasted 2 weeks, with assessments administered at baseline and 2 weeks after the intervention. The 2-week intervention period was selected to evaluate short-term feasibility, acceptability, and preliminary efficacy while minimizing participant burden. Intervention participants were also invited to complete an optional open-ended feedback interview. The study was approved by the Weill Cornell Medicine Institutional Review Board, and informed consent was obtained from all participants electronically via the Research Electronic Data Capture (REDCap) e-Consent framework. The trial protocol is provided in Supplement 1, and the study followed the Consolidated Standards of Reporting Trials (CONSORT) reporting guideline.

### Participants

The sample included individuals 18 years or older who provided primary care to a family member or friend with mild to moderate ADRD living in the community. Caregivers completed an online survey to gather eligibility and self-reported demographic information (eg, sex, race and ethnicity), the categories for which were derived from the National Institute on Aging’s Clinical Research Operations & Management System reporting guidelines. Race and ethnicity were categorized as American Indian or Alaska Native, Asian, Black or African American, Hispanic or Latino, Native Hawaiian or Other Pacific Islander, White, and multiracial; these data were included because they were important demographic information needed to adequately characterize the study sample and explore potential racial and ethnic differences with respect to the intervention. This information provides insight into the generalizability of study findings across diverse caregiver populations. Disease stage was assessed with a single item asking whether care recipients exhibited mild (eg, memory loss with preserved independence), moderate (eg, worsened cognitive and/or behavioral impairments), or severe (eg, profound impairment necessitating extensive care) symptoms.

Caregivers and their care recipients were also required to be fluent in English, reside within the US, and have computer and internet access. Caregivers were excluded if they were not primary ADRD caregivers, if they self-reported a cognitive impairment, or if their care recipients resided in long-term care or exhibited advanced cognitive impairment that could impede LMH-4-DCP use. Enrollment occurred between November 6, 2024, and May 15, 2025. Participants were recruited via an online platform connecting participants with institutional review board–approved studies, social media advertisements, and announcements at the Duke–University of North Carolina Alzheimer’s Disease Research Center.

### Protocol and Contact Schedule

Interested participants completed an online eligibility survey via REDCap. After providing electronic consent, participants were randomized and completed online baseline assessments. Randomization occurred through REDCap, in which a permuted block design that was stratified by sex and race and ethnicity generated 2:2 allocations within blocks of 4 to ensure demographic balance across conditions. Study personnel were unable to view or edit randomization tables once uploaded. Participants were blinded to condition assignment.

Participants received group-specific access to securely register for LMH-4-DCP, with login and instructions distributed via email. Training materials included condition-specific video tutorials (videos, PDF manuals) providing an overview of the platform and onboarding process. Participants were instructed to log on 3 times weekly (6 times total) to ensure adequate exposure to the assigned content. Email reminders were sent at 1 week to encourage adherence and offer technical support.

Participants were compensated $50 per assessment. Interview completers received an additional $25.

### Study Conditions

Participants were randomly assigned to the intervention or attention control group. Each group accessed 1 of 2 versions of the LMH-4-DCP platform, allowing for consistent engagement while isolating the impact of reminiscence-based content.

#### Intervention Group

Intervention participants were given unrestricted access to the full LMH-4-DCP platform, which included 3 interactive “rooms” (screens) to support dyadic reflection and storytelling. The Reminiscence Room featured 2 image-based activities: Memory Lane, a digital scrapbook where participants upload images with captions, and the Wall of Fame, a gallery showcasing photos to facilitate collaborative memory sharing. The Writing Room offered 2 writing-based activities: This Is Your Life included guided prompts tied to life stages (early childhood) with a photograph-upload option and a journaling tool with open-ended prompts (“What is your favorite memory of your mother?”) to support life review and narrative meaning-making. The Reading Room provided a Resource Dictionary for general resources, Tips and Tricks with caregiver resources, and an Information Station containing downloadable scientific publications.

#### Attention Control Group

Control participants accessed a restricted version of LMH-4-DCP, without RT features, containing activities that matched the intervention group’s time commitment and interface interaction. Control participants selected a virtual home environment to personalize the user interface, completed journal entries using neutral (non-RT), present-focused prompts (eg, “What’s on your mind today?”), and were given the same caregiving resources as intervention participants.

### Outcomes

#### Feasibility

Recruitment feasibility was calculated using the proportion of family caregivers who consented, declined, and were ineligible at screening. Consent rates were calculated as the number of participants who consented divided by the number of eligible individuals. Completion rates were the number of completed assessments divided by the total assigned (68 × 2 assessments = 136). Attrition rates were calculated as the number of noncompleters divided by the total number enrolled (n = 68).

Website feasibility was assessed using the 10-item System Usability Scale (SUS)^26^ at follow-up. Items were rated on a 5-point Likert scale (1 indicates strongly disagree; 5, strongly agree). Scores were converted to range from 0 to 100. Higher scores indicated greater usability.

#### Acceptability

Acceptability was assessed at follow-up via an 8-item survey measuring satisfaction, perceived usefulness, and ease of use. Participants rated their level of agreement on a 5-point Likert scale (1 indicates strongly disagree; 5, strongly agree). Intervention participants also responded to 5 open-ended items exploring aspects of the intervention perceived as engaging, helpful, or needing improvement.

#### Predeath Grief

The predeath version of the PGD (12-item Prolonged Grief Revised [PG-12-R])^5,27^ measure, administered at baseline and follow-up, included 12 items asking caregivers to report the frequency of grief symptoms (eg, yearning, bitterness) due to their care recipient’s terminal condition. Responses were rated on a 5-point Likert scale ranging from 1 (not at all) to 5 (several times a day or overwhelmingly). Total scores ranged from 12 to 60; higher scores indicated greater predeath grief (Cronbach α = 0.88).

#### Relationship Quality

The 6-item Relational Deprivation Scale^3^ measured perceived relational loss resulting from the care recipient’s illness. Response options ranged from 1 (not at all) to 4 (completely), with higher scores indicating poorer relational quality (Cronbach α range, 0.67 to 0.77). The 15-item Mutuality Scale^28^ evaluated positive aspects of caregiving; responses ranged from 0 (not at all) to 4 (a great deal), with higher scores reflecting greater mutuality (Cronbach α = 0.90). The 8-item Relationship Rewards Scale asked caregivers to evaluate their relationship before (4 items; Cronbach α = 0.81) and after (4 items; Cronbach α = 0.84) the care recipient’s ADRD diagnosis.^29^ Responses ranged from 1 (never) to 4 (always), with higher scores indicating greater relational rewards.

### Statistical Analysis

Data were analyzed from August 1 to 31, 2025. Descriptive statistics were computed for sociodemographic characteristics across the total sample and by group. Feasibility and acceptability ratings among intervention participants were summarized descriptively. Outcomes (predeath grief, relationship quality) were assessed for within- and between-group differences. Paired sample *t* tests evaluated intervention group changes from baseline to 2 weeks after the intervention. Independent sample *t* tests examined differences between the intervention and control groups at follow-up. Mean change scores for each outcome were calculated by subtracting posttest from pretest values and compared across groups. Cohen *d* was calculated to estimate effect sizes (0.2 indicated small; 0.5, medium; and 0.8, large). Missing data were handled via listwise deletion. Analyses were conducted using SPSS, version 29.0 (IBM Corp). Statistical significance was defined as *P* < .05, and all hypothesis tests were conducted using 1-sided tests in accordance with the study’s directional hypotheses.

## Results

### Sample

Sixty-eight participants enrolled in the pilot study (34 per condition). The mean (SD) age was 49.4 (13.7) years (age range, 23-90 years). Fifty-four participants (79.4%) were female and 14 (20.6%) were male. In terms of race and ethnicity, 1 caregiver (1.5%) was American Indian or Alaska Native, 4 (5.9%) were Asian, 12 (17.6%) were Black, 9 (13.2%) were Hispanic or Latino, 1 (1.5%) was Native Hawaiian or Other Pacific Islander, 48 (70.6%) were White, and 2 (2.9%) were multiracial. More than half of caregivers (39 [57.4%]) were adult children of patients with ADRD. The mean (SD) age of the care recipients was 75.4 (9.3) years. Table 1 provides participant characteristics for the full sample and across study conditions. No differences in sociodemographic characteristics emerged between groups.

**Table 1.** Participant Characteristics

Characteristic	Study group, No. (%)^a^		
	All (N = 68)	Intervention (n = 34)	Attention control (n = 34)
Caregiver age, mean (SD), y	49.4 (13.7)	47.1 (12.8)	51.9 (14.3)
Care recipient age, mean (SD), y	75.4 (9.3)	75.5 (9.8)	75.3 (8.9)
Caregiver sex			
Female	54 (79.4)	27 (79.4)	27 (79.4)
Male	14 (20.6)	7 (20.6)	7 (20.6)
Care recipient sex			
Female	34 (50.0)	18 (52.9)	16 (47.1)
Male	34 (50.0)	16 (47.1)	18 (52.9)
Caregiver marital status			
Married or partnered	39 (57.4)	18 (52.9)	21 (61.8)
Separated or divorced	7 (10.3)	5 (14.7)	2 (5.9)
Never married	18 (26.5)	9 (26.5)	9 (26.5)
Widowed	4 (5.9)	2 (5.9)	2 (5.9)
Caregiver race and ethnicity			
American Indian or Alaska Native	1 (1.5)	0	1 (2.9)
Asian	4 (5.9)	2 (5.9)	2 (5.9)
Black	12 (17.6)	6 (17.6)	6 (17.6)
Hispanic or Latino	9 (13.2)	2 (5.9)	7 (20.6)
Native American or Other Pacific Islander	1 (1.5)	1 (2.9)	0
White	48 (70.6)	23 (67.6)	25 (73.5)
Multiracial	2 (2.9)	2 (5.9)	0
Caregiver educational level			
Some college or less	12 (17.6)	7 (20.6)	5 (14.7)
Associate’s degree	8 (11.8)	3 (8.8)	5 (14.7)
Trade or vocational school	3 (4.4)	2 (5.9)	1 (2.9)
Bachelor’s degree or higher	45 (66.2)	22 (64.7)	23 (67.6)
Caregiver employment status			
Employed full-time	30 (44.1)	14 (41.2)	16 (47.1)
Employed part-time	10 (14.7)	6 (17.6)	4 (11.8)
Unemployed	9 (13.2)	3 (8.8)	6 (17.6)
Retired	10 (14.7)	5 (14.7)	5 (14.7)
Homemaker	8 (11.8)	6 (17.6)	2 (5.9)
Preferred not to respond	1 (1.5)	0	1 (2.9)
Care recipient dementia type			
Alzheimer disease	29 (42.6)	16 (47.1)	13 (38.2)
Vascular dementia	8 (11.8)	3 (8.8)	5 (14.7)
Other dementia (Lewy body, Parkinson, frontotemporal)	16 (23.5)	8 (23.5)	8 (23.5)
No formal diagnosis	15 (22.1)	7 (20.6)	8 (23.5)
Caregiver kinship to care recipient			
Spouse	10 (14.7)	6 (17.6)	4 (11.8)
Adult-child	39 (57.4)	21 (61.8)	18 (52.9)
Other extended family	15 (22.1)	7 (20.6)	8 (23.5)
Friend	4 (5.9)	0	4 (11.8)

^a^ Randomization was stratified to assign equal numbers of males and females to each study condition; therefore, sex did not differ between groups.

### End Points

#### Feasibility

##### Recruitment Feasibility

Of 174 completed prescreenings, 106 met inclusion criteria. The remainder (n = 68) were ineligible because the respondent was not a primary ADRD caregiver (n = 39) or the care recipient resided in residential care (n = 5), had advanced dementia (n = 10), or did not speak English (n = 8). Four caregivers were excluded due to self-reported cognitive impairments, and 2 lacked internet access.

Of 106 eligible caregivers, 34 could not be reached and 4 declined participation. Sixty-eight participants (64.2%) consented and were randomized to the intervention (n = 34) or control condition (n = 34). No differences emerged among eligible participants who did vs did not enroll. Prior to baseline, 1 intervention participant was lost-to-follow-up and 1 control participant withdrew due to lack of interest. Eleven participants (6 in the intervention group and 5 in the control group [16.2%]) were lost to follow-up after allocation. Sixty-six (33 per group) baseline assessments were completed and 54 participants (27 per group) completed 2-week follow-ups. No significant differences emerged on sociodemographic or key study variables between participants who completed assessments vs dropped out.

Across the sample, 58 participants (85.3%) logged into LMH-4-DCP at least once and 120 of 136 assigned assessments were completed, yielding a completion rate of 88.2%. Completion was equivalent across groups (60 assessments per condition). Attrition, calculated as the number of participant noncompleters (n = 14) divided by total number enrolled (n = 68), was 20.6% for the sample and was evenly distributed across groups (7 noncompleters per group divided by 34 enrolled). The CONSORT diagram is shown in the Figure. The findings support the feasibility of intervention delivery, with 13 of 15 participants (86.7%) older than 60 years of age logging onto LMH-4-DCP at least once; 6 of 15 (40.0%) meeting the prescribed engagement target (≥6 logins); and 10 of 15 (66.7%) completing required assessments.

**Figure.**
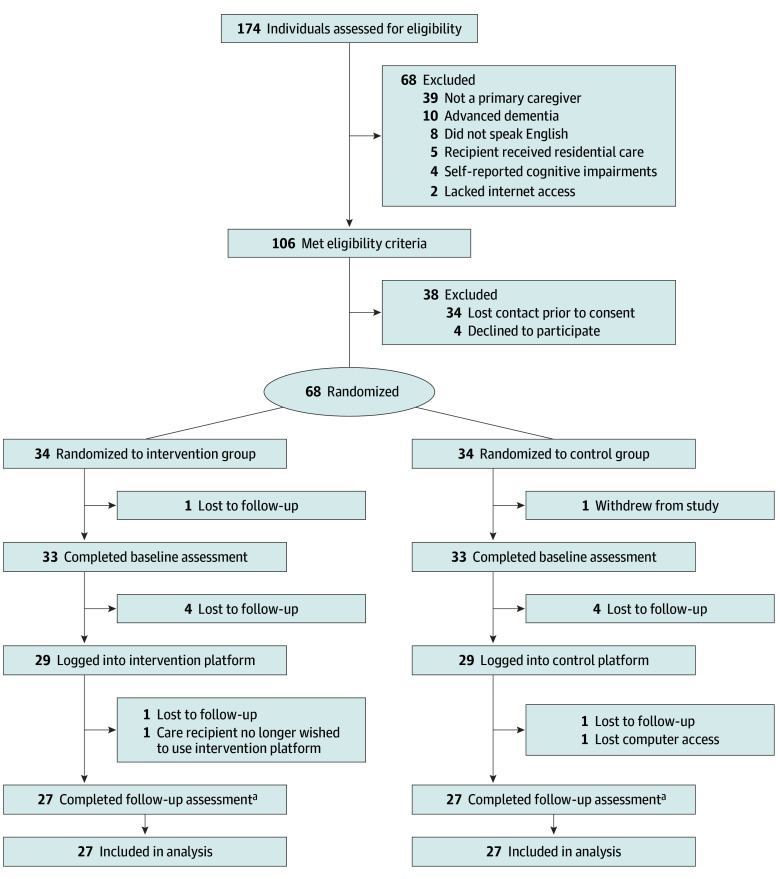
Study Flow Diagram ^a^An optional follow-up interview was completed by 5 patients.

##### Website Feasibility

Using 68 as a threshold for adequate usability, the mean (SD) SUS score for LMH-4-DCP participants (n = 27) was 73.61 (6.05). Most intervention participants agreed or strongly agreed that LMH-4-DCP was easy to use (20 of 27 [74.1%]) and had well-integrated features (21 of 27 [77.8%]), and they felt confident using the platform (19 of 27 [70.4%]).

#### Acceptability

The mean (SD) score for the 8-item acceptability survey was 32.85 (6.05) of a maximum of 40, indicating a moderate-to-high degree of acceptability (eAppendix in Supplement 2 provides item-level responses). Open-ended responses showed that participants valued LMH-4-DCP for its ability to strengthen dyadic engagement, with reminiscence, journaling, and photograph sharing rated as the most beneficial features. Suggestions for improvement included enhanced audiovisual features (n = 5 [ie, games, music, recording]), functionality (n = 3 [ie, “I would’ve liked a more seamless experience”]), and design refinements (n = 3 [ie, “It was tricky to know where to click next”]). Many participants (n = 19) reported no suggested improvements. As one participant shared, “I feel this will be a good technology bridge for people. I think it will interest both care partners and the individual.”

### Preliminary End Points

#### Within Participants

Change scores during a 2-week period were calculated at the individual level as posttest minus baseline values. In the intervention group (n = 27), the mean (SD) PG-12-R score significantly decreased from baseline (25.08 [10.28]) to after testing (21.78 [8.26]), reflecting a mean (SD) reduction of 3.29 (8.64; *t*_26_ = −1.98; *P* = .03) with a small to moderate effect (Cohen *d =* −0.38). While not statistically significant, current relationship quality increased from a mean (SD) of 11.94 (3.49) before testing to 12.48 (2.19) at after testing (*P* = .29) (Table 2).

**Table 2.** Paired Sample *t* Tests for Study Variables at Baseline and After Testing in the Intervention Group^a^

Variable	Score, mean (SD)		*t* Test	*P* value	Cohen *d*
	Baseline	After testing			
Predeath grief					
PG-12-R^b^	25.08 (10.28)	21.78 (8.26)	−1.98	.03	−0.38
Relationship quality					
Mutuality^c^	50.21 (16.55)	52.90 (12.73)	0.28	.39	0.05
Relational deprivation^d^	14.15 (4.43)	15.07 (5.18)	0.96	.17	0.18
Preillness relationship rewards^e^	12.93 (2.67)	12.80 (2.37)	−0.84	.20	−0.16
Current relationship rewards^e^	11.94 (3.49)	12.48 (2.19)	0.54	.29	0.10

Abbreviation: PG-12-R, 12-item Prolonged Grief Revised.

^a^ Includes 27 participants. Missing data handled using listwise deletion.

^b^ Scores range from 12 to 60, with higher scores indicating greater predeath grief.

^c^ Measured using the 15-item Mutuality Scale; scores for each item range from 0 to 4, with higher scores indicating greater mutuality.

^d^ Measured using the 6-item Relational Deprivation Scale; scores for each item range from 0 to 4, with higher scores indicating poorer relational quality.

^e^ Measured using the 8-item Relationship Rewards Scale; scores for each item range from 1 to 4, with higher scores indicating greater relational rewards.

#### Between Participants

No significant differences emerged at follow-up (Table 3). Current relationship rewards at 2 weeks were higher among intervention participants (mean [SD], 12.48 [2.19]) than controls (mean [SD], 11.55 [2.53]), with a mean (SD) difference of 0.93 (0.64) that did not indicate significance (*t*_51.17_ = 0.75; *P* = .08; Cohen *d* = 0.39).

**Table 3.** Independent Sample *t* Tests Comparing Intervention and Control Groups at Baseline and Follow-Up^a^

Variable	Intervention group		Control group		*t* Test	*P* value	Cohen *d*
	No. of participants	Mean (SD) score	No. of participants	Mean (SD) score			
**PG-12-R: predeath grief** ^b^							
Baseline	34	25.08 (10.28)	34	23.38 (8.09)	0.76	.23	0.18
Follow-up	27	21.78 (8.09)	27	23.85 (9.19)	−0.87	.19	−0.24
**Mutuality** ^c^							
Baseline	34	50.21 (16.55)	34	52.35 (13.20)	−0.59	.28	−0.14
Follow-up	NA	NA	NA	NA	NA	NA	NA
**Relational deprivation** ^d^							
Baseline	34	13.38 (4.60)	34	14.18 (4.76)	−0.26	.39	−0.06
Follow-up	27	15.07 (5.18)	27	14.07 (4.56)	0.75	.23	0.21
**RRS: preillness** ^e^							
Baseline	34	12.94 (2.75)	34	13.11 (2.72)	−0.27	.39	−0.07
Follow-up	27	12.62 (2.37)	27	12.37 (2.57)	0.39	.35	0.11
**RRS: current relationship** ^e^							
Baseline	34	11.94 (3.49)	34	12.15 (2.43)	−0.28	.39	−0.07
Follow-up	27	12.48 (2.19)	27	11.55 (2.53)	1.44	.08	0.39

Abbreviations: NA, not applicable; PG-12-R, 12-item Prolonged Grief Revised; RRS, Relationship Rewards Scale.

^a^ Missing data handled using listwise deletion.

^b^ Scores range from 12 to 60, with higher scores indicating greater predeath grief.

^c^ Measured using the 15-item Mutuality Scale; scores for each item range from 0 to 4, with higher scores indicating greater mutuality.

^d^ Measured using the 6-item Relational Deprivation Scale; scores for each item range from 0 to 4, with higher scores indicating poorer relational quality.

^e^ Scores for each item range from 1 to 4, with higher scores indicating greater relational rewards.

To evaluate between-group differences, individual-level change scores were compared using independent sample *t* tests (Table 4). Predeath grief decreased in the intervention group (mean change, −3.30) and slightly increased in the control group (mean change, approximately 0.47), yielding a between-group mean (SD) difference of −3.60 (1.51) points favoring the intervention with a moderate effect size (*t*_40.67_ = −1.90; *P* = .03; Cohen *d* = −0.51). Changes in relationship quality did not significantly differ between groups.

**Table 4.** Change in Secondary Outcomes From Baseline to After Testing for Intervention and Control Groups^a^

Variable	Mean (SD) change (after testing − baseline)	*t* Test	*P* value	Cohen *d*
PG-12-R^b^	−3.60 (8.64)	−1.90	.03	−0.51
Relational deprivation^c^	0.70 (5.04)	0.49	.31	0.13
Mutuality^d^	NA	NA	NA	NA
RRS: preillness^e^	0.22 (1.84)	0.43	.33	0.12
RRS: current^e^	0.63 (2.51)	1.04	.15	0.28

Abbreviations: NA, not applicable; PG-12-R, 12-item Prolonged Grief Revised; RRS, Relationship Rewards Scale.

^a^ Missing data handled using listwise deletion.

^b^ Scores range from 12 to 60, with higher scores indicating greater predeath grief.

^c^ Measured using the 6-item Relational Deprivation Scale; scores for each item range from 0 to 4, with higher scores indicating poorer relational quality.

^d^ Due to technical difficulties with the data collection platform, the mutuality scale was not administered to control group participants, and thus this variable was excluded from this analysis.

^e^ Scores for each item range from 1 to 4, with higher scores indicating greater relational rewards.

## Discussion

This pilot RCT examined the feasibility, acceptability, and preliminary efficacy of the LMH-4-DCP platform for predeath grief among caregivers of patients with ADRD. The findings support the feasibility of intervention delivery, with 86.7% of participants older than 60 years of age logging onto LMH-4-DCP at least once; 40.0% meeting the prescribed engagement target (≥6 logins); and 66.7% completing required assessments. LMH-4-DCP’s usability was supported by a mean SUS score of 73.61, exceeding the standard benchmark of 68.^26^ Although 10 participants (14.7%) did not log on during the study, qualitative feedback from open-ended acceptability items suggested that those who engaged with the platform perceived it as useful.

Adequate adherence is supported by the 88.2% study assessment completion rate. The observed attrition rate of 20.6% during the 2-week period is in line with or lower than attrition reported in other web-based ADRD caregiver interventions.^30,31^ Attrition (n = 14) was driven by practical and contextual barriers, including loss of contact (n = 11), internet access challenges (n = 1), and variability in caregiver or care recipient engagement (n = 2). These findings highlight the importance of designing flexible, low-burden digital interventions that account for dyadic variability. Future studies may benefit from strategies to support sustained engagement, such as enhanced technical support and additional check-ins to address challenges.

Acceptability of LMH-4-DCP was strong, with high ratings pertaining to participant satisfaction, enjoyment, and ease of use. These findings suggest that LMH-4-DCP could improve psychosocial well-being among caregivers of patients with ADRD. Analysis of mean change scores from baseline to follow-up showed that predeath grief significantly decreased in the intervention compared with the control group, with a moderate effect size (Cohen *d* = −0.51).

These findings within the ADRD predeath context complement the broader postdeath grief literature, which supports using memory recall and reflection to facilitate bereavement adjustment.^32,33^ While research has identified links between emotional regulation difficulties and grief severity,^34,35^ RT is associated with reduced depression, anxiety, and enhanced cognitive and emotional regulation.^15,16,36,37,38^ Thus, the potential for RT to promote cognitive reframing may explain the predeath grief decrease among intervention group participants.

Overall, results underscore RT as a promising approach to promote meaningful interaction to address the psychosocial voids that emerge from ADRD caregiving.^18^ Conceptually, RT overlaps with interventions (eg, dignity therapy), facilitating narrative reconstruction, legacy building, and existential integration in the context of illness.^39,40^ While these approaches are typically clinician facilitated and individually focused, LMH-4-DCP emphasizes dyadic, collaborative life review using a digital, self-guided format to promote accessibility and preserve core narrative and meaning-centered elements common to other therapeutic approaches.

Although not significant, current relationship quality in the intervention group improved compared with the control group after testing. According to the interdependence model of communal coping, meaningful engagement in shared activities can buffer against stress, enhance quality of life, and strengthen relationship quality,^19,20^ with evidence indicating that better relationship quality is associated with better dyadic outcomes.^21,22,23^ Closer relationships thus represent a promising, modifiable intervention target to improve dyadic mental health and well-being. Given the small sample size and 2-week assessment intervals, an adequately powered and longer intervention period may detect more robust effects. Nonetheless, our findings suggest that enhancements to LMH-4-DCP—particularly features that explicitly foster the relationship (eg, interactive elements, turn-taking in questions)—may strengthen effects on relationship quality.

### Limitations

Several study limitations should be considered. The small sample limited statistical power to detect between-group differences, thus limiting our originally planned analysis of linear mixed effects modeling. Additionally, assessment of care recipient cognitive status and/or ADRD stage relied on single-item caregiver-reported indicators; future work should incorporate objective measures of cognition. Participants were predominantly female, White, English speaking, highly educated, and drawn from urban areas, limiting generalizability to broader ADRD caregiver populations (eg, rural, underresourced caregivers). The eligibility criteria required that participants have device and internet access, and recruitment efforts were largely virtual, which likely yielded a sample with greater digital literacy, further limiting generalizability.

The 2-week intervention period may have been insufficient to yield measurable changes in relationship quality, warranting further investigation into the duration needed to observe improvements. Furthermore, we did not evaluate whether meeting the minimum login requirements differentially impacted outcomes. Because this study prioritized feasibility, it was not powered to examine dose-response or identify activity components most strongly associated with change. Future trials should explore the types and amount of engagement required to derive benefit. Additionally, formal adjustment was not undertaken for multiple tests, increasing risk for type I error; thus, findings should be interpreted with caution. While results are hypothesis generating, LMH-4-DCP’s preliminary efficacy on predeath grief supports the need for a well-powered RCT focused on minimizing attrition and the need for refined features to further promote relationship quality.

## Conclusions

This pilot RCT of a digital RT intervention for ADRD family caregivers found that LMH-4-DCP was feasible, was highly acceptable, and demonstrated preliminary efficacy in reducing predeath grief, with potential improvements in current relationship quality. These findings highlight RT as a promising therapeutic activity to reduce predeath grief and enhance relationships, providing initial support for LMH-4-DCP as a scalable and accessible behavioral intervention for ADRD care pairs. Its web-based format and minimal staff requirement enhances its potential for clinical implementation in health care (eg, memory clinics) and community settings as a low-cost, strengths-based activity for ADRD caregiver–care recipient dyads

## Notes

Supplement 1.
Trial Protocol

Supplement 2. **eAppendix.** Acceptability Questionnaire Item-Level Responses

Supplement 3.
Data Sharing Statement
