# Supplementary material for: Digital Reminiscence for Predeath Grief Among Family Caregivers of Patients With Dementia: A Pilot Randomized Clinical Trial
Source: JAMA Netw Open. 2026 Apr 22;9(4):e268278. doi: 10.1001/jamanetworkopen.2026.8278 (PMC13103805; doi:10.1001/jamanetworkopen.2026.8278)
Supplement: Supplement 1. — Trial Protocol [file jamanetwopen-e268278-s001.pdf]

Supplementary Materials S.2.: Study IRB Approval and Protocol

Weill Cornell Medicine
Research Integrity

Institutional Review Board

Notification of Approval - Amendment

12-May-2025

To: Holly Prigerson PhD

Amendment to Study Title: The Living Memory Home: Reducing Grief and Improving Relationships between Home-based Patients with ADRD and Their Family Caregivers

**WRG Submission Number**	23-07026251-15
**Approval Date of Amendment**	12-May-2025
**Protocol Expiration Date**	n/a
**Post Approval Monitoring Due**	18-Aug-2025
**Risk Level**	Minimal Risk
**FWA Number**	FWA00000093

This amendment was reviewed and approved by the WCM IRB in accordance with ethical standards and the requirements of the Code of Federal Regulations on the Protection of Human Subjects (45 CFR 46, and if applicable 21 CFR 50, 56, 312, and 812).

Additional modifications or changes to this protocol must be submitted to the WCM IRB for review and approval prior to implementation. Once all research activities have been completed, a request for closure must be submitted.

As part of the WCM IRB's Post Approval Monitoring program, a Post-Approval Monitoring Annual Report (PAM-AR) will be required annually. Please submit your post-approval monitoring report at least 60 days before the due date listed above.

The principal investigator must report any adverse events or unanticipated problems resulting from this study to the WCM IRB. The principal investigator remains responsible for following all pertinent ethical and legal guidelines as well as WCM policies.

In order to access the approved documents tab, click on the Submission Page: Submission Page. The Approved Documents tab will appear on the top bar next to the Linkages and Submissions tab.

If you have any questions, please contact Human Research Compliance at 646-962-8200 or email irb@med.cornell.edu.

Sincerely,

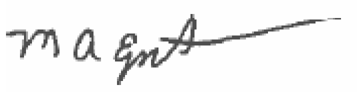

Melissa A. Epstein, PhD, MBE, CIP
Executive Director
Human Research Protection and Compliance
Weill Cornell Medicine

This is official correspondence from the WCM IRB Office

research.weill.cornell.edu

TITLE: The Living Memory Home: Reducing Grief and Improving Relationships between Home-based Patients with ADRD and Their Family Caregivers

IRB Protocol #: 23-07026251
Version Date: 04/04/2025
Funding Source(s): National Institute on Aging (NIA)

Principal Investigators:

Holly G. Prigerson, PhD
420 East 70th Street
4th Floor, Room 423
Cornell Center for Research on End-of-Life Care
New York, NY 10021
(212) 746-1374
Email: hgp2001@med.cornell.edu

Francesca B. Falzarano, PhD
University of Southern California Leonard Davis
School of Gerontology
Los Angeles, CA 90089
Email: falzaran@usc.edu

Co-Investigators:

Paul K. Maciejewski, PhD
525 E 68th St, Box 39,
1414 Baker Pavilion
New York, NY 10065
Email: Pam2056@med.cornell.edu

Lavender (Wan Jou) She, PhD
Cornell Center for Research on End-of-Life
Care New York, NY 10021
Email: wjs2004@med.cornell.edu

Joe Miller
Cornell Center for Research on End-of-Life
Care New York, NY 10021
646-962-7591
Email: jom4013@med.cornell.edu

Annabelle Greenfield
University of Southern California Leonard Davis
School of Gerontology
Los Angeles, CA 90089
Email: ag69282@usc.edu

Sydney Saviano
Cornell Center for Research on End-of-Life Care
New York, NY 10021
sys4002@med.cornell.edu

Sindhu Kolla
Cornell Center for Research on End-of-Life
Care New York, NY 10021
Sik4009@med.cornell.edu

Statistician: Dr. Maciejewski will serve as the study's statistician

Participating Sites:

- Site Name
- Weill Cornell Medicine Center for Research on End-of-Life Care
  PI: Holly G. Prigerson, PhD
  Center Phone: (646) 962-9910
  Email: hgp2001@med.cornell.edu

1. Table of Contents

Table of Contents

2. Confidentiality Statement 4
3. List of Abbreviations 4
4. Protocol Summary 6
4.1.1 Study Population 6
4.2 Study Objectives 9
4.2.1 Objectives 9
4.2.2 Hypotheses / Research Questions 10
5. Background and Significance 10
6. Study Design and Methods 11
6.1 Overall Design 11
6.2 Proposed Features of LMH-4-DCP 1.0 12
6.3 Interviews, Surveys, and/or Observations 16
6.3.1 Phase 2: 17
6.3.2 Screening Assessments 18
6.3.3 Semi-structured Interview 18
6.3.4 Phase 2 18
6.3.5 Survey Measures 18
6.3.6 Semi-Structured Feasibility Interview 20
6.3.7 C. Location 20
6.3.8 D. Personal Identifiers 20
7. Study Design 20
7.1 Study Population 20
7.2 Inclusion Criteria 21
7.3 Exclusion Criteria 21
7.4 Strategies for Recruitment and Retention Recruitment (Phase 1) 21
7.4.1 Recruitment (Phase 2) 22
7.4.2 Retention 23
7.4.3 Compensation 23
8. Registration Procedures 24
8.1 Subject Registration (WCM only) 24
9. Study Procedures 24
9.1 Schedule of Assessments 24
10. Data Reporting / Regulatory Considerations 25
10.1 Data Collection 25
10.1.1 REDCap 25
10.2 Regulatory Considerations 26
10.2.1 Institutional Review Board/Ethics Committee Approval 26
10.2.2 Ethical Conduct of the Study 26
10.2.3 Informed Consent 26
10.2.4 Compliance with Trial Registration and Results Posting Requirements 27
10.2.5 Record Retention 27
11. Statistical Considerations 28

12. References 28

2. Confidentiality Statement

This document is confidential and is to be distributed for review only to investigators, potential investigators, consultants, study staff, and applicable independent ethics committees or institutional review boards. The contents of this document shall not be disclosed to others without written authorization from WCM.

3. List of Abbreviations

**ADRD**	Alzheimer's Disease and Related Dementias
**APIM**	Actor Partner Interdependence Model
**Co-I**	Co-Investigator
**CR**	Care Recipient
**CTSA**	Clinical and Translational Science Award
**CTSC**	Clinical Translational Science Center
**DCP**	Dementia Care Pair
**Duke-UNC ARDC**	Duke University School of Medicine and University of North Carolina at Chapel Hill Alzheimer's Disease Research Collaborative
**FAQ**	Frequently Asked Questions
**FDAAA**	Food and Drug Administration Amendments Act
**FDAMA**	Food and Drug Administration Modernization Act
**GCP**	Good Clinical Practice
**HIPAA**	Health Insurance Portability and Accountability Act of 1996
**ICF**	Informed Consent Form
**ICH**	International Council for Harmonisation
**IEC**	Independent Ethics Committee
**IP**	Investigational Product
**IRB**	Institutional Review Board

**LDAP**	Lightweight Directory Access Protocol
**LMH**	Living Memory Home
**LMH-4-DCP**	Living Memory Home-4-Dementia Care Pairs
**NIA**	National Institute on Aging
**PGD**	Prolonged Grief Disorder
**PG-12**	Prolonged Grief-12
**PI**	Principal Investigator
**PSSUQ**	Post-Study System Usability Questionnaire
**PwD**	Person with Dementia
**RCT**	Randomized Control Trial
**REDCap**	Research Electronic Data Capture
**RT**	Reminiscence Therapy
**SPMSQ**	Short Portable Mental Status Questionnaire
**SUS**	System Usability Scale
**UBACC**	University of California, San Diego Brief Assessment of Capacity to Consent
**WCM**	Weill Cornell Medicine
**WRG-CT**	Weill Research Gate– Clinical Trials

4. Protocol Summary

Please provide a brief protocol summary.

Full Title: The Living Memory Home: Reducing Grief and Improving Relationships between Home-Based Patients with ADRD and Their Family Caregivers

Short Title: Living Memory Home-4-Dementia Care Pairs (LMH-4-DCP)

Principal Investigator: Holly G. Prigerson & Francesca B. Falzarano

Study Description: The goal of this two-phased study is to 1) adapt the Living Memory Home (LMH), an online bereavement resource, to be a pre-loss, reminiscence-based web intervention for caregivers of patients with early to moderate stages of dementia; and 2) conduct a pilot randomized controlled trial (RCT) to examine intervention feasibility and acceptability. **Phase 1** of this study gathered insights to inform the development and adaptation of LMH for dementia family caregiver-care-recipient dyads (hereby referred to as "care pairs") using semi-structured interviews with current and/or bereaved family caregivers, mild-to-moderate stage Alzheimer's Disease and Related Dementias (ADRD) patients, as well as dementia care professionals/subject matter experts. Participant feedback on proposed website modifications and features informed the iterative refinement of the website and intervention for Phase 2 activities. **Phase 2** will explore the feasibility, acceptability, and preliminary effects of LMH-4-DCP versus an attention control condition on outcomes including pre-loss grief and care pair relationship quality. Participants include dementia family caregivers who will engage with their care-recipient on the LMH-4-DCP platform three times a week over a two-week period. Assessments of outcomes will be conducted through surveys at baseline and at the two-week follow-up to measure the LMH-4- DCP's impact.

Sample Size: **Phase 1.** Semi-structured interviews, n=30
Phase 2. Pilot RCT, n=70 dementia family caregivers (n=35 randomized to LMH-4-DCP; n=35 randomized to attention control condition)

Enrollment: **Phase 1** will enroll 30 relevant experts: n=20 current/bereaved dementia family caregivers and persons with dementia (PwD); dementia care experts, n=10.
Phase 2 will enroll 70 dementia caregivers.

4.1.1 Study Population:

Phase 1: Interviews (n=30): Current and/or bereaved primary family caregivers of community-dwelling PwD, PwD in the mild-to-moderate stages demonstrating capacity to consent, and ADRD subject matter experts.

Phase 2. 1) <u>Surveys:</u> Quantitative assessments conducted at baseline and two-week follow-up via REDCap; 2) <u>Interviews:</u> (Optional) Qualitative, observational interviews facilitated by study personnel to gather feedback on the LMH-4-DCP web application and intervention.

Enrollment Period: 07/01/2024 – 04/30/2025

Study Design:

Phase 1.

Relevant experts, including family caregivers, PwD with capacity to consent, and dementia-care experts will participate in a one-time semi-structured interview facilitated by a trained member of the research study staff. Participants will be asked to participate in a *Design Probe,* whereby they will view and provide feedback on a PowerPoint slide deck containing feature design mock-ups/wireframes, descriptions of proposed website modifications, and new features applicable for dementia care dyads to be incorporated into LMH-4-DCP. They will be queried for structured feedback on the website's content, aesthetics, structure, flow, and features, they will also be asked about technology proficiency and knowledge of/willingness to use web-based reminiscence therapy. Participants will also be asked to provide self-reported demographic information (e.g., age, gender, race, ethnicity, marital status) as well as information about the PwD (e.g., dementia type/severity, hours of care provided per week, care activities/intensity).

Phase 2.

A pilot RCT in which n=70 dementia caregivers will be randomized to either the LMH-4-DCP intervention (n=35), with both reminiscence and non-reminiscence features, or the attention control condition, which consists of a version of the platform that excludes reminiscence-based activities (n=35). This study aims to assess the feasibility and acceptability of the LMH-4-DCP application and explore its effects on pre-loss grief and care pair relationship quality. The total targeted sample size is n=70 participants. The participant (the caregiver) will complete baseline assessments comprised of surveys assessing mental health, relationship quality, quality of life, technology proficiency, caregiver burden, and social support. The participant in both conditions will be provided with training and instruction on how to use the website and will be instructed to interact with the web application 3 times per week for two weeks.

Reminiscence-based activities include journaling about memories, uploading photos to a digital scrapbook and virtual picture frames, and drafting an autobiographical-style "book". Non-reminiscence activities involve journaling current experiences and emotions, and accessing dementia-care resources including scholarly articles, national and virtual resources, and informal resources such as blogs, videos, and articles related to dementia caregiving. At the conclusion of the two-week participation period, the caregiver will complete follow-up assessments that include the survey measures administered at baseline, in addition to assessments of feasibility and acceptability. Participants will be given the opportunity to participate in an optional virtual interview to evaluate LMH-4-DCP's feasibility and provide feedback on their experience using the platform.

Facilities Enrolling Participants:

Phases 1 & 2. Participants will also be recruited via WCM's Center on Aging, the Duke University School of Medicine and University of North Carolina at Chapel Hill Alzheimer's Disease Research Collaborative (Duke-UNC ADRC), and the Leonard Davis School of Gerontology at the University of Southern California. Project collaborators will assist in outreach efforts to connect prospective participants with the WCM team. Participants may also respond to announcements via researchmatch.org and emails/flyers distributed at community organizations (or through their email listservs) serving the target population: primary ADRD caregivers of community dwelling PwD. Further, a description of, and portal of entry to, the study will be posted on the Cornell Center for Research on End-of-Life Care website.

Study Duration:

Phases 1 & 2. Approximately 18 months total.

Participant Duration:

Phase 1.

1) One online survey assessing background and demographic characteristics lasting approximately 10-20 minutes.
2) One 30-45-minute semi-structured interview.

Phase 2.

1) One online survey assessing background and demographic characteristics (lasting approximately 5-10 minutes).
2) A pre-intervention baseline assessment comprised of online surveys (lasting approximately 15-20 minutes).
3) Completion of LMH-4-DCP web-based study activities for approximately one hour, three times per week for two weeks.
4) One virtual post-intervention follow-up survey (lasting approximately 10-15 minutes)

5) One optional semi-structured interview on Zoom (post-intervention) to provide feedback on the LMH-4-DCP website and application, (lasting approximately 30 minutes).

Primary Objective: **Phase 1.** Adapt the Living Memory Home (LMH) online bereavement resource to be a pre-loss RT based activity tailored for dementia care dyads to engage in together based on feedback from relevant experts.

Phase 2. Examine feasibility, acceptability, and preliminary efficacy in a pilot RCT of a reminiscence-based intervention for family caregiver-PwD dyads to record and reflect on meaningful memories to address family caregivers' pre-loss grief and enhance relationship quality. Only caregivers will act as study participants.

Exploratory Objectives: **Phase 1.** Not applicable

Phase 2: Conduct exploratory analysis on caregiver psychosocial deprivations (e.g., sense of belonging; role confusion; social isolation) as potential mediating influences on the association between LMH-4-DCP's use on pre-loss grief and relationship quality.

Primary Endpoints: **Phase 1.** Outcomes will include structured feedback from participants on the initial iteration of LMH-4-DCP's usability, including content and language; design, and aesthetics (colors, feature creep); content of journal prompts; newly proposed features/activities; potential problems; suggestions for website improvement.

Phase 2. The pilot RCT aims to evaluate LMH-4-DCP's feasibility, acceptability, and explore preliminary effects on caregivers' pre-loss grief and their relationship quality with their PwD.

Secondary Endpoints: **N/A.**

4.2 Study Objectives

The objectives of this project are to adapt, refine, and pilot test a reminiscence-based online platform for caregiver-PwD dyads that: a) the caregiver will probe and record the PwD's life story; and b) provide collaborative RT activities that are expected to reduce pre-loss grief and improve the care pair relationship. Our micro-sociological theory of adaptation to loss[1] posits that addressing psychosocial deprivations associated with a significant interpersonal loss will improve one's adjustment. For ADRD caregivers, psychosocial voids include a restructuring of their relationship, diminished sense of belonging and feeling appreciated, role confusion, self-esteem, respect for and nurturance of the PwD, and social isolation.

4.2.1 Objectives

Our specific objectives will be completed in two phases.

- Phase 1: Adapt and iteratively refine the LMH online platform to include dyadic online RT activities for caregivers and PwD in the mild-to-moderate stages of the disease course. We will engage expert informants to comment and provide feedback on the tool's proposed features. The tool will be refined based on participant feedback.

- In Phase 2: A pilot RCT will be conducted with dementia family caregivers to determine feasibility, acceptability, and preliminary effects on caregiver pre-loss grief and care pair relationship quality. We will also conduct exploratory analyses on potential mediators (e.g., sense of belonging) on caregivers' psychosocial outcomes.

4.2.2 Hypotheses / Research Questions

We hypothesize that engaging with LMH-4-DCP's features will reduce ADRD caregiving-related psychosocial deprivations and, thereby, reduce pre-loss grief while improving the caregiver's relationship with the PwD. Through exploratory analysis, this study will provide an empirical test of our micro-sociological theory of adaptation to loss in the context of dementia.

5. Background and Significance

With roughly 6.7 million families in the United States impacted by ADRD, 83% of individuals with ADRD are cared for by family members, largely in their own homes.[2] The rising prevalence of ADRD necessitates a growing demand for family caregivers to provide care for these individuals, emphasizing its significance as a critical public health concern. The inexorable decline and loss of the PwD's memories, identity, and functioning makes ADRD among the most dreaded and feared diseases.[3] Not only is the PwD helpless to prevent memories from slipping away, but the family members who care for them navigate a dramatic restructuring of their relationship, often fearing that the PwD's identity and life story may be forgotten and lost to posterity.[4,5] Deterioration of an individual's cognitive functioning, including increasing impairments in memory recall and behavioral symptoms associated with dementia, are hallmarks of the disease's progression. Ultimately, the PwD transitions to a state of complete dependency, relying on mostly unpaid family members or friends to provide for their basic needs.

Beyond the roles associated with direct care provision, family members must navigate ambiguous losses – in which the PwD is physically present, but increasingly mentally absent. The loss of an existing relationship, the shared identity with a close significant other, and the role changes that accompany disease progression exacerbates feelings of pre-loss grief – a yearning for the PwD as they were prior to the disease, longing for their prior relationship (e.g., relational deprivation), loneliness, sorrow, and role confusion. Pre-loss grief can be detrimental for ADRD caregivers, who are responsible for the lion's share of care and decision making on behalf of the PwD. Even in dementia's early stages, 44-71% of ADRD caregivers report high levels of pre-loss grief, with higher rates in caregivers who reside with the PwD.[6-8]

A major loss caregivers confront is the loss of their relationship with the significant other, what some refer to as "relational deprivation".[9-11] Caregivers not only miss the identity of the PwD, but also their once existing relationship, creating psychosocial voids akin to those experienced in bereavement. Consistent with this hypothesis, we have shown pre-loss grief in caregivers and dying patients is linked, and that caregiver grief declines when the "care pair" engages in shared activities such as the patient's advance care planning.[12] These findings suggest that addressing caregivers' psychosocial deprivations has potential for reducing pre-loss grief and improving the care pair relationship (**Figure 1**). According to the Interdependence Model of Communal Coping[13,14] engaging in activities that are meaningful to the dyad may reduce the negative effects of stress on coping, enhance quality of life, and improve the quality of the dyadic relationship. This work suggests the promise of a dyadic approach to improving caregiver mental health as well as the care pair's relationship.

Figure 1. Conceptual Model of Psychosocial Deprivations Targeted by the Living Memory Home to Reduce Grief & Improve Relationship Quality

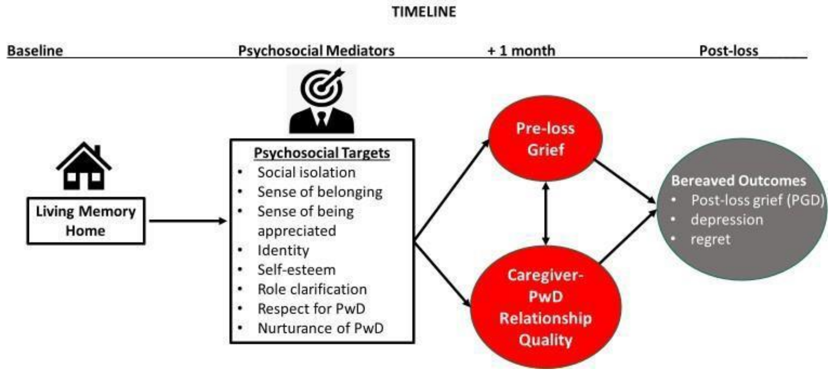

Further, pre-loss grief has been largely absent from most conceptualizations of the dementia caregiver stress process. Not only can pre-loss grief be more severe than post-loss grief, but pre-loss grief has been shown to be synchronized with patients' grief[12] and is a predictor of poor bereavement outcomes.[5,15-19] Pre-loss grief is also linked to the caregivers' relationship to the dying patient.[19-23] While caregiver pre-loss grief is cross-sectionally associated with depression and anxiety,[24] poor coping and quality of life,[25] and worse end-of-life decision making,[26,27] it also predicts future poor bereavement outcomes, including Prolonged Grief Disorder (PGD), one of the strongest predictors of suicidal ideation among caregivers.[28-30] This empirical literature underscores the need to target the reduction of pre-loss grief among family caregivers of PwD providing care in their home. Thus, we hypothesize that targeting the reduction of pre-loss grief in dementia family caregivers is expected to reduce risk of pre- and post-loss distress, disease, and dysfunction. Our micro sociological theory of adaptation to loss[31] suggests that caregivers' well-being benefits from filling the psychosocial voids created by a significant interpersonal loss. In the context of ADRD, such voids may be filled by engaging in meaningful activities enabling caregivers to share memories with the PwD, recall happy memories, discuss proud moments, as well as providing an opportunity to show affection and respect for the PwD.

Reminiscence therapy (RT) uses cues to trigger recall of significant life events, a technique often used in clinical long-term care settings. Because roughly 66% of PwD reside in the community with their primary caregiver, we hypothesize that a dyadic RT-based online program facilitated by the family caregiver with the engagement of the PwD will help address the psychosocial deprivations that are common in dementia family caregiving – ultimately reducing caregivers' feelings of pre-loss grief and improving the perceived quality of the care pair's relationship.

This project, funded by the NIA (R21AG077144), will leverage our existing online LMH bereavement resource (IRB #: 1810019629) to adapt, develop, refine, and evaluate LMH-4-DCP's feasibility, acceptability, and ability to improve targeted outcomes (e.g., caregiver pre-loss grief, relationship quality). LMH-4-DCP is expected to provide positive social interactions, promote closeness, identity, pride, and mutual appreciation.

6. Study Design and Methods

6.1 Overall Design

Phase 1. Adaptation and refinement of LMH-4-DCP version 1.0.

1. Develop wireframes and mock-ups with proposed features to be incorporated into LMH-4-DCP Version 1.0; Prepare design probe to solicit feedback from participants.
2. A PowerPoint presentation of the proposed features and modifications will be presented to participants to guide feedback discussions. The interview guide for Phase 1 semi-structured interviews can be found in the *Supplementary Materials*.

6.2 Proposed Features of LMH-4-DCP 1.0

i. Home Selection
   - The current LMH requires users to select a home and landscape upon login. LMH-4-DCP will provide additional home styles (e.g., beach house, urban townhouse). Each different home style will be accompanied by three rooms (reminiscence, writing, and reading) of the same theme. Currently, users are directed to a “memorial space” windowsill, but this will become a “Reminiscence Room” in LMH-4-DCP.

ii. Reminiscence Room:
   - Writing Down Memory Lane
     a. Family caregivers and their care recipients (CR) can utilize this scrapbook-style feature to reminisce on memories. By uploading photos to their digital scrapbook and adding captions/descriptions, they can come together to cherish and reflect on special moments.
   - Wall of Fame
     a. Family caregivers can mount pictures of the care pair’s choosing, hanging images of people and things of which they are proud. The images uploaded to the scrapbook in “*Writing Down Memory Lane”* will be automatically uploaded to the *“Wall of Fame”* feature as options for photos to mount on the wall. In addition, the pair will have the option to upload any other photos of their choosing from their own photo library. This will encourage the pair to reminisce over memories, since the pair will be able to look through their photos and choose the photos that they would like to mount on the wall of their “Reminiscence Room”.

iii. Writing Room
   - This is Your Life
     a. To record the PwD’s memories, caregivers will act as an autobiographer documenting the PwD’s “*This is Your Life*” review of people, places, and things of most special significance. *Should the PwD express frustration during this exercise, the caregiver will be instructed to turn to other activities as the goal is to trigger positive remembrances and not upset the PwD.*
     b. Potential *This is Your Life* prompts may include:
        i. Where were you born?
        ii. Where did you grow up, go to school?
        iii. Where did you live as a young adult? Where did you get married, raise your family?
        iv. Did you work outside the home? If yes, what type of work did you do?
        v. Did you take vacations? What were your favorite trips/vacations?

vi. Are there accomplishments you are most proud of? Things you would like others to know about you?

vii. What are you good at? What are you not so good at?

viii. What were some difficult challenges you faced in your life? How did you navigate them?

- Journaling
  - a. As part of the features provided, structured journal prompts will be available for individuals with ADRD patients and their caregivers. These prompts aim to encourage thoughtful reflection and initiate conversations between the pair.
  - b. Potential journal prompts include:
    1. How are you feeling today?
    2. Do you have any plans for the day? If yes, what are they?
    3. What are things that you like to do?
    4. If you could do anything now what would it be?
    5. Who are the people closest to you? Who do you like? Who do you dislike?
    6. These days, who or what brings you the most joy?
    7. Do you have hobbies?
    8. What sports do you like?
    9. What type of music do you like?
    10. Do you have a favorite food? Foods that you dislike?

iv. Reading Room

- Provides care pairs with resources and tips on coping with dementia and caregiving through three different features embedded into this room:
  - a. Resource Dictionary
    - i. A comprehensive resource guide that includes an extensive list of organizations, helplines, and supportive services for both caregivers and PwD. This feature serves as a valuable reference, providing pairs with a centralized and easily accessible repository of resources that can offer assistance, guidance, and support across various domains.
  - b. Tips and Tricks
    - i. A place to find valuable advice and techniques to support caregivers in navigating their responsibilities. It will include a variety of media such as podcasts, videos, and articles.
  - c. Information Station
    - i. A hub of condensed educational resources sourced through the available scientific literature for care pairs to understand and learn more about the research in dementia, dementia care, caregiving, and more.

3. Recruit 30 relevant experts, including concurrent/bereaved family caregivers and persons with dementia (PwD) (N=20) and dementia care experts (N=10).
   - a. Informed e-consent via REDCap will be obtained prior to participation.

   b. Relevant expert feedback will be obtained via semi-structured interview "Design Probes" after participants are presented with a PowerPoint slide deck of proposed features. Relevant experts will be prompted to provide structured feedback on the website's content, language, flow (sequencing of activities, speed/timing), aesthetics (color scheme, feature creep, sounds, clutter), usability, and new and proposed features. *See Supplementary materials.*
4. The team will convene to review relevant expert feedback, generate and discuss a list of proposed modifications, and votes will be taken to decide on modifications to iteratively refine the user interface.

Phase 2. Iterative refinement and pilot testing of LMH-4-DCP Version 2.0

1. Report feedback from semi-structured interviews conducted in Phase 1 to refine proposed features of LMH-4-DCP 2.0
2. Launch LMH-4-DCP Version 2.0 in two iterations for the intervention and control groups. The intervention group will be assigned to a version of LMH-4-DCP that includes all website features (both RT and non-RT activities). Caregivers in the control group will be given access to a limited version of LMH-4-DCP that includes only non-reminiscence-based elements. Additional details regarding the final website features are presented in Table 1.
3. Recruit 70 caregivers to participate in an RCT of LMH-4-DCP.
   a. Interested participants will complete a brief screening survey via REDCap to determine their eligibility and gather basic demographic information. Unlike Phase 1, only the family caregiver will be consented and enrolled into the study, since we are not collecting any information of the person with dementia
   b. Based on the screening survey, study staff will email participants to inform them of their eligibility status and next steps.
   c. Eligible participants will be asked to schedule times to meet with the study staff, where the family caregiver will complete the consent process. After the participants complete their consent meetings they will be randomly assigned via REDcap to the intervention (n=35; RT group) or control arm (n=35; non-RT group) in a 1:1 ratio.
   d. Research staff will send baseline surveys via email to gather quantitative data on measures including mental health, social support, and relationship quality. Some of the survey batteries for caregivers will ask questions about the PwD that the caregiver can report themselves. For details on the specific measures each caregiver will take, please refer to **Table 2**. Each individual survey battery will be sent to the caregiver via email.
   e. Participants will receive training on the use of LMH-4-DCP through virtual instruction, including PDFs and audiovisual tutorials. They will also have access to instructional materials available on the LMH-4-DCP website. The control group will receive instructions specifically tailored to the limited version of the website and activities they will be completing.
   f. The participant and their care-recipient will interact with their web application for two weeks, logging on three times per week for one-hour each. Weekly reminders to log in and complete study activities will be sent via email. Data on weekly website engagement, such as number of logins, feature usage, and time spent on each activity, will be gathered. Additionally, participants will receive a 1-week check-in call on Zoom with a member of the study team to ask any questions or share any concerns.
   g. After two weeks of using LMH-4-DCP, research staff will send caregivers a follow-up survey via email, tailored to each study group. These surveys will include the

same measures as the baseline surveys, along with additional items to assess the platform's feasibility and usability. See **Table 4** for assessment schedule.

h. Participants will be invited to participate in an optional semi-structured interview with a study team member, lasting about 30 minutes, to give feedback on the LMH-4-DCP web application and intervention.

Table 1. Refined Features Included in LMH-4-DCP 2.0

**Feature Name**	**Description**	**RT (Intervention)**	**Non-RT (Control)**
**Reminiscence Room**			
Writing Down Memory Lane	A digital scrapbook where users can upload photos and are asked to reflect and reminisce on experiences and events. Family caregivers will facilitate documenting the PwD's response.	•	
Wall of Fame	Users upload and choose photos and respond to generated prompts. Caregivers will be focused on both the PwD's meaningful accomplishments (e.g., graduation) and/or significant people in the PwD's life (e.g.,<br>family photos).	•	
**Writing Room**			
This is Your Life	Caregivers will serve as biographers, interviewing PwD using prompts to document their major milestones, ultimately resulting in a chronological 'Life Story' across early childhood, adolescence, young, middle, and older adulthood. User entries will be compiled to produce a "Book of Your Life," chronologically presenting major "chapters" of the PwD's life. Users have the option to export and save as PDF.	•	
Journaling (non-RT group)<br>• For caregiver<br>• For care-recipient	Structured prompts will be used for<br>participants to reflect and		•

	document current feelings and experiences, hobbies, and/or any other relevant circumstances or scenarios occurring in the present.		
Journaling (RT group)<br>• For caregiver<br>• For care-recipient	The journal will contain structured prompts that will be used for caregivers to facilitate and document current feelings and experiences, and hobbies, as well as reminiscence-activities that focus on recalling past events.	•	
**Reading Room**			
Resource Dictionary	A comprehensive list of formal home and community-based organizations (HCBOs) both locally and nationally. Includes organizations, helplines, and supportive communities.		•
Tips & Tricks	Informal sources of support, such as podcasts, YouTube videos, social media accounts, and articles from the popular press, provide caregivers with helpful tips, educational content, and information to more effectively navigate their roles.		•
Information Station	Access to latest scientific research and advancements via articles, publications, and help guides/toolkits in ADRD, family caregiving, etc.		•

6.3 Interviews, Surveys, and/or Observations

Phase 1 of this study is an observational semi-structured interview study of 30 ADRD caregivers, PwD exhibiting capacity to provide consent, and key organizational and content relevant experts, with expertise in providing care and/or supporting dementia family caregivers. Participants will complete a single semi-structured interview lasting approximately 45-60 minutes to provide feedback on LMH-4-DCP's newly proposed features. Participants will also be asked to provide self-reported demographic information (e.g., age, gender, race, ethnicity, marital status, relationship to the PwD), questions assessing technology proficiency, as well as information about the PwD (e.g., dementia type/severity, hours of care provided per week, care activities). Relevant experts will complete self-report demographic questionnaires (e.g., age, race, ethnicity, job position, specialty). For additional details regarding Phase 1 assessments, refer to **Table 3.**

A sample of 30 relevant experts will consist of caregivers, eligible PwD, and dementia care providers with expertise in supporting PwD and caregivers, ensuring representation from different training and specialties (e.g., clinicians, social workers, visiting nurses) and representative sampling by sex and race. Research/content experts will be identified based on expertise in dementia and/or caregiving. Participation is virtual (e.g., telephone, Zoom). Participants will be compensated $50 via Amazon gift cards or equivalent.

6.3.1 Phase 2:

In **Phase 2** of this study, a pilot RCT will be conducted to determine feasibility, acceptability, and preliminary effects of Version 2.0 of the LMH-4-DCP web-application, in which a sample of 70 dementia caregivers will be randomized to the LMH-4-DCP intervention (n=35) or to the attention control condition (LMH-4-DCP without reminiscence activities; n=35). Each caregiver is caring for a person with mild-to-moderate dementia. Feedback obtained during Phase 1 interviews was used to inform the iterative refinement of the application's interface and the development of study activities during Phase 2.

Once a participant indicates interest in enrolling in the study, a member of the study team will share a REDCap survey link to determine their eligibility. This screening will ask family caregivers to provide self-reported demographic information (e.g., age, gender, race, ethnicity, marital status, relationship to the PwD), as well as information about the PwD (e.g., dementia type/severity hours of care provided per week, PwD's demographics).

If a participant is deemed eligible, they will be invited to meet with a member of the study team to review the study's procedures and subject guidelines and provide informed consent. Participants will then complete baseline assessments after informed consent is provided. For additional details regarding Phase 2 assessments, refer to **Table 4**.

Following baseline assessments, enrolled caregivers in the intervention condition will receive specialized tutorials and instructional materials from a member of the research staff on the use of LMH-4-DCP online tool and the intervention instructions. Control arm participants will receive training on and access to a limited version of LMH-4-DCP, without reminiscence-focused activities. Control group participants will be assessed on the same schedule as those in the intervention arm (baseline and 2-weeks). All participants will receive user tutorials and have access to a FAQ webpage. Additionally, they will be provided with email and telephone contacts to address any technical difficulties they might encounter during the study.

After the two-week study period is complete, intervention group caregivers will be invited to participate in an optional 30-minute semi-structured interview to provide feedback on their experience using the LMH-4-DCP web-application.

B. Study Instruments

Phase 1
Sociodemographic Characteristics

- ***Background and Demographics*****:** Caregivers will be asked to provide self-reported demographic information (e.g., age, gender, race, ethnicity, marital status) as well as information about the PwD (e.g., dementia type, hours of care provided per week). Relevant experts will complete self-report demographic questionnaires (e.g., age, race, ethnicity, job position, specialty). With participant consent, all interviews will be audio-recorded and transcribed. Participants will still be allowed to take part in the study if they do not give their consent to be recorded.

6.3.2 Screening Assessments

- ***University of California, San Diego Brief Assessment of Capacity to Consent (UBACC):*** The UBACC is validated in ADRD samples to assist investigators in identifying research participants who may require a more comprehensive evaluation of their decision-making abilities and/or additional support before they can be enrolled in the study.[34,35] PwD participants must exhibit capacity to consent by scoring ≥ 9 on the UBACC to ensure adequate understanding of the nature of their participation.
- ***The Short Portable Mental Status Questionnaire (SPMSQ)***: The SPMSQ is a questionnaire consisting of 10 items that has been specifically created to serve as a tool for identifying and assessing the severity of cognitive impairment.[32] To be eligible to participate, family caregivers must have normal cognitive functioning (as indicated by a score of less than 3 on the SPMSQ. If the family caregiver deems the person with ADRD for whom they care as capable of participating, the PwD scores below 8 on the SPMSQ (indicating mild to moderate cognitive impairment).

6.3.3 Semi-structured Interview

- ***Feedback for Proposed Features.*** Feedback on proposed features will be gather during a 45-minute semi-structured interview led by a trained interviewer from the study team. The interviewer will present a design probe of the proposed features and user interfaces to the participant, where they will be asked to provide feedback on various aspects of the LMH-4-DCP web application.

6.3.4 Phase 2
Sociodemographic Characteristics

- ***Background and Demographics*****:** Caregivers will be asked to provide self-reported demographic information (e.g., age, gender, race, ethnicity, marital status) as well as basic information about their experiences as a caregiver (e.g., dementia type, hours of care provided per week). Caregivers will also be asked to provide basic demographic information about the PwD. With participant consent, all interviews will be audio-recorded and transcribed. Participants will still be allowed to take part in the study if they do not give their consent to be recorded.

6.3.5 Survey Measures

- A list of survey measures to be administered to participants is presented in **Table 2**.

Table 2. LMH-4-DCP RCT Intervention "Measurement Map"

Domain	Instrument Name	Description

Sociodemographic Characteristics	N/A	Age, sex, race/ethnicity, education, geographic location, income, CR functional status and dementia-related problem behaviors.
Technology Proficiency	Computer Proficiency Questionnaire (CPQ) (modified)[36]	16-item scale assessing older adults' proficiency with computer use.
Identity Confusion	Erikson's Psychosocial Stage Inventory (EPSI)[37]	12-item questionnaire assessing identity confusion based on Erikson's stages of psychosocial development.
Belonging/Appreciation	Interpersonal Needs Questionnaire (INQ)[38]	15-items assessing the extent to which individuals feel connected to and valued by others.
	Interpersonal Support Evaluation List (ISEL)[39]	40-item questionnaire measuring the availability and quality of social support that individuals perceive they have.
Role Confusion	Role Captivity[40]	3-item inventory measuring the extent of role confusion and the psychological impact of feeling confined to a specific role.
Rewards	Positive Aspects of Caregiving (PAC)[41]	11-item questionnaire evaluating the positive experiences and rewards associated with caregiving.
Social Support	Lubben Social Network Scale-6 (LSNS-6)[42]	6-item inventory assessing the availability and quality of social relationships and support systems.
Depression	Patient Health Questionnaire (PHQ-9)[43]	9-item scale assessing the severity of depressive symptoms.
Burden	Zarit Burden Inventory-12 (ZBI-12)[44]	12-item scale measuring caregiver burden, including emotional, physical, and financial stress.
Anxiety	General Anxiety Disorder-7 (GAD-7)[45]	7-item scale assessing the severity of anxiety symptoms.
Quality of Life	The World Health Organization Quality of Life (WHO-QoL Bref) (modified)[46]	A modified 5-item scale assessing quality of life across different domains.
Primary Outcomes		
Feasibility	Number of log-ins per participant out of total required (minimum= 6); participant retention	# of website logins, time spent in each activity, feature usage; Proportioned of caregivers who 1) consents; 2) declined participation; 3) did not meet eligibility criteria
Usability	System Usability Scale (SUS)[47,48]	10-items assessment of the usability of a system, helping identify areas for
		improvement.

Acceptability	Satisfaction, acceptability	Survey and open-ended items assessing usability, engagement, acceptability, suggestions for improvement
Secondary Outcomes		
Pre-Loss Grief	Pre-loss Grief-12 (PG-12)[49,50]	12-item measure assessing severity of pre-loss grief symptoms experienced by individuals who are caring for or have a loved one with a terminal illness
Relationship Quality	Relational Deprivation[51]	6-item inventory measuring the perceived relationship loss that caregivers experience as a result of their relative's illness.
	Mutuality[52]	15-item questionnaire assessing the positive aspects of caregiving relationships, emphasizing reciprocal positive feelings and interactions.
	Relationship Rewards Scale (RSS)[53]	8-item questionnaire evaluating the perceived rewards and benefits derived from personal relationships.

6.3.6 Semi-Structured Feasibility Interview

- ***LMH-4-DCP Post-Intervention Feedback Interview.*** This optional qualitative interview (lasting approximately 30-minutes) will gather feedback and participant reactions to the LMH-4-DCP web application and intervention. Participants will be administered series of open-ended questions posed by a trained interviewer from the study team. See enclosed Phase 2 interview guide for interview prompts.

6.3.7 C. Location

The entire study will be conducted online, with no in-person components. Throughout the study duration, all collected data will be carefully de-identified and securely stored on the secure server of the Cornell Center for Research on End-of-Life Care. Only the authorized investigators involved in the study will be granted access to the data.

6.3.8 D. Personal Identifiers

Each participant will receive a unique participant ID, ensuring that their survey responses remain confidential and de-identified. These records will be securely stored in protected locations for at least three years, in compliance with the timeframes specified by WCM and HIPAA policies.

7. Study Design

7.1 Study Population

Phase 1: Current or bereaved dementia family caregivers and PwD (n= 20); relevant experts (n=10).

Phase 2: Caregivers (N=70)

7.2 Inclusion Criteria:

Caregivers are eligible to participate if they are: a) a primary source of care for a family member or friend in the early to moderate stages of dementia and who speaks English fluently, b) 18 years of age or older, c) English- speaking, d) able to use the internet and has internet access, and d) residing in the United States (Phases 1 and 2).

PwD are eligible to participate if: a) the caregiver deems the PwD able, b) the PwD scores < 8 on the SPMSQ, c) they demonstrate capacity to consent to a low-risk study as indicated by a score of ≥9 on the UBACC, and d) they reside in the United States (Phases 1 only).

Dementia experts are eligible to participate if they: a) are over the age of 18-years-old, b) have experience with development and implementation of resources in dementia care organizations and/or have experience serving families impacted by dementia, c) are English speaking, d) reside in the United States (Phase 1 only).

7.3 Exclusion Criteria:

Caregiver exclusion criteria: a) is not the primary family caregiver of the PwD; b) under the age of 18-years-old, c) exhibits cognitive impairment d) does not speak English as a primary language and e) (Phase 2 only) the caregiver deems the PwD's dementia too severe to participate in the application activities. (i.e. cannot have a coherent conversation)

Relevant expert exclusion criteria: a) are under the age of 18-years-old; b) does not actively provide care/support to PwD or family caregivers; c) does not possess expertise in dementia and/or caregiving; d) does not speak English as a primary language (*Phase 1 only*)

7.4 Strategies for Recruitment and Retention Recruitment (Phase 1)

We plan to recruit 30 participants, including concurrent/bereaved family caregivers and persons with dementia (PwD) (N=20) and dementia care experts (N=10). Study participants will be recruited from WCM's Center for Research on End-of-Life Care and Center on Aging. Recruitment efforts will include posting flyers on social media platforms such as Instagram, Twitter, LinkedIn, and Facebook. We will also contact previous study participants who agreed to be contacted for future research opportunities and use researchmatch.org, a national database of individuals interested in research participation, to disseminate Institutional Review Board (IRB)-approved study information to potential participants. Interested individuals from any of these sources will contact the WCM study team via email for further information and next steps.

We selected ADRD caregivers to be part of this group of participants because this is the sample that will be targeted to participate in the LMH-4-Dementia Care Pairs' (LMH-4-DCP) intervention in Phase 2 of the current study. Additionally, while current caregivers provide a "real-time" perspective on caregiving, bereaved caregivers can provide a retrospective look back on their experiences as caregivers, with the advantage of having had time to reflect on the experience

after the PwD's death. We consider both perspectives invaluable for informing the design and adaptation of the LMH-4-DCP intervention.

Dementia care experts (n=10) with experience in the development and implementation of resources in dementia organizations and experience with supporting ADRD patients and caregivers will be nominated via targeted sampling, ensuring adequate representation by age, sex, and race, and from different training and specialties including social workers, palliative care physicians, nurses, and caregiving advocates.

7.4.1 Recruitment (Phase 2)

We plan to recruit 70 caregivers from WCM's Center on Aging, (NYC, NY) with outreach support from Duke-UNC ADRC and USC. Potential caregiver participants will be identified by our recruitment partner Dr. Heather Whitson (site-PI and Co-I, Duke-UNC ADRC) and/or members of their staff at their respective sites.

Duke-UNC ADRC and USC's Los Angeles Family Caregiver Center will assist in facilitating distribution and outreach of our study materials through existing site resources including the North Carolina Registry for Brain Health and the Duke Dementia Family Support Program. The project's research assistant will work directly with our site collaborators weekly to identify potential participants who may be eligible and interested in participation. Additionally, outreach staff will identify eligible participants through their pool of caregivers, as well as through the distribution of study materials via their newsletters, outreach teams, and other programs. We will ask current caregivers if the ADRD patient for whom they care would be capable of responding meaningfully to questions about the LMH-4-DCP intervention; we will only approach those patients whom the family caregiver considers willing and able to determine the PwD's eligibility. We will also assess the PwD's capacity to consent prior to enrollment in this study.

Recruitment of Dementia Caregivers: Outreach support at Duke-UNC ADRC and USC will distribute study information. Potential participants will be identified through the WCM Center for Research on End-of-Life Care web portal, as well as through referrals and outreach efforts from Duke UNC-ADRC and USC. Additionally, researchmatch.org will be utilized to recruit prospective participants. Flyers will be distributed at community organizations, senior centers, social media, and community events. Convenience and snowball sampling methods will be employed to identify suitable organizations for this minimal risk study. We will not recruit participants without speaking to and obtaining permission from the director, assistant director, and/or program director at community organizations. Participants will be made aware that the study is entirely voluntary, and that participation will not impact their relationship with, or the services received, at their respective centers. Community organizations may distribute study flyers so that interested persons can reach out directly to research staff at WCM. Research staff may also call or email previous participants who have expressed interest in participating in future study opportunities and through community outreach (e.g., public health fairs). These potential participants opted-in for future contact and were/are also given the opportunity to opt-out of any future contact.

We will collect basic identifying information for all participants who indicate an interest in participating in the study, including name, address, phone number, and email so that we may contact them to explain the nature of the study, determine eligibility, and schedule a time to obtain informed consent. Prospective participants will be contacted by WCM study team members to verify interest, review eligibility criteria, obtain informed consent, and schedule an interview at a time, manner, and location most convenient for the participant (e.g., videoconference, and/or telephone). If the participant agrees to participate, according to their preference, they will provide

their consent via REDCap’s e-consent framework, or they will have the option of being mailed 2copies of the consent form with a pre-addressed, stamped return envelope or emailed 1 copy of the consent form. The study team will follow up with potential participants to confirm they received the consent form, review the informed consent, and answer any questions the participants may have. The participant will be guided to send one consent form back and keep a copy for their records. Participants may return the signed consent form via mail, fax, or email back (through secure WCMC file transfer).

For those who want more time to think about their decision to sign the ICF, we will thank them for considering participation, and inform them that we will follow up with a call the following week to discuss their decision. If the signed ICF is sent to study staff by mail, staff will sign upon receipt of this document and enter the date the consent form was received.

Following study completion, participants will be asked if they are willing to permit the research team to retain their contact information to stay informed about future study participation opportunities.

7.4.2 Retention

Phase 1. Given that ADRD caregivers, PwD, and relevant experts will take part in a single interview for study activities, there is no major concern regarding participant retention. However, we will ensure that participants understand the overall objectives of the study and recognize the value of their contributions, promoting their active engagement. To minimize any potential burden on participants, we will work with them to schedule interviews at convenient times, considering their preferences for interview modality (such as in-person, telephone, or Zoom teleconferencing) and location. We will provide them with relevant content and a manageable list of questions to guide their feedback. The interviews are expected to last between 45 to 60 minutes.

Phase 2. Caregivers will participate in the LMH-4-DCP intervention for 2 weeks (approximately one month between completion of their eligibility screening to completion of the optional feedback interview). Participants will undergo a baseline assessment, expected to take about 10-15 minutes. Following this, they will be randomized into either group and engage in assigned activities three times per week for two weeks. After completing the activities, participants will complete a follow-up survey within a week of the program's conclusion to evaluate the study's end-point outcomes. Participants will also have the option to take part in an optional Zoom interview with a member of the study team to gather final feedback and assess feasibility.
Interviews will be scheduled to be convenient for participation in terms of date/time. Further, the research staff is trained to be attentive and sensitive to participants’ status (e.g., fatigue, distress) and concerns, and will communicate with them throughout the duration of their participation to see how they are doing. Therefore, we will have a “check-in” email and/or Zoom or telephone call 1 week after first the participant first logs into the web application to evaluate how the subject’s participation is going, answer questions, and strategize about addressing any challenges (technical or psychological) they may have encountered. These practices have been shown in our prior studies (MH 121886) to strengthen participants’ commitment to remain in the study and reduce study attrition.

7.4.3 Compensation:

Participants in **Phase 1** will receive $50 compensation upon interview completion. Phase 2

participants will receive $50 compensation for completing the baseline and $50 for the 2-week follow-up assessment (for a total of $100). Participants who opt to take part in the feedback interview will be compensated with an additional $20. Compensation will be provided via Amazon gift cards or equivalent.

8. Registration Procedures

8.1 Subject Registration (WCM only)

Subjects will be registered within the WRG-CT as per the standard operating procedure for Subject Registration.

9. Study Procedures

9.1 Schedule of Assessments

Table 3. Phase 1 LMH-4-DCP Assessment Schedule

Measure	Pre-Study	Phase 1 Study Visit
Background and Demographics [Relevant Experts, CG, PwD]	•	
Capacity to consent [PwD]	•	
Cognitive screening [PwD]	•	
Feedback for Proposed Features [Relevant Experts, CG, PwD]		•

Table 4. Phase 2 LMH-4-DCP Assessment Schedule

Measure	Pre-Screen	Baseline	2-Week
Background and Demographics	•		
Computer Proficiency Questionnaire (modified)		•	•
Erikson's Psychosocial Stage Inventory		•	•
Interpersonal Needs Questionnaire		•	•
Interpersonal Support Evaluation List		•	•
Role Captivity		•	•
Positive Aspects of Caregiving		•	•
Lubben Social Network Index		•	•
PHQ-9		•	•
ZBI-12		•	•
GAD-7		•	•
WHO-QoL Scale		•	•

PG-12		•	•
Relationship Rewards		•	•
Relational Deprivation		•	•
Mutuality		•	•
Satisfaction & Acceptability			
System Usability Scale			•
LMH-4-DCP Feedback Interview [intervention groups only]			•

10. Data Reporting / Regulatory Considerations

10.1 Data Collection

The data collection plan for this study is to utilize REDCap to capture all data for all enrolled subjects. All data obtained will be de-identified.

Phase 1: Initially, we will gather personal information from individuals who express interest in participating in the study. This includes their name, address, phone number, and email. The purpose of collecting this information is to contact them, provide an explanation of the study, assess their eligibility, and schedule a time to obtain informed consent. Once enrolled, each participant will be assigned a unique identifier number, which will be used to link their data.

Phase 2: In this phase, we will collect basic identifying information from caregivers who express interest in participating in the study. This will include their name, address, phone number, and email, which will be used to contact them, explain the purpose of the study, assess eligibility, and obtain informed consent. Upon enrollment, each caregiver will be assigned a unique identifier number to ensure accurate linkage of their data.

All individuals who enroll in the study will be assigned a unique identifier number to which their data will be linked. A document linking study identifier numbers to identifying information will be stored in a single master document on a secure WCM server that can only be accessed via password-protected computers that are stored in locked offices by credentialed study personnel. All coded data will be stored on a secure WCM server in a folder that can only be accessed by credentialed study personnel on password protected computers in locked offices or on a duo-security, password protected REDCap database.

10.1.1 REDCap

REDCap (Research Electronic Data Capture) is a comprehensive data management software system that is fully supported by the Weill-Cornell Medical Center CTSC. It offers a range of features including the creation of customized, secure data management systems with web-based data-entry forms, reporting tools, and robust security measures such as user and group-based privileges. It utilizes institution LDAP authentication for user access and maintains a complete audit trail of data manipulation and export activities. REDCap is hosted on CTSC-owned servers, which are backed up regularly and support encrypted (SSL-based) connections. It is part of a

larger consortium led by Vanderbilt University CTSA, ensuring ongoing development, enhancements, and support on a national level.

10.2 Regulatory Considerations

10.2.1 Institutional Review Board/Ethics Committee Approval

Before initiating the study, the Investigator will ensure compliance with local regulations by covering all legal aspects and obtaining approval from the appropriate regulatory bodies. At each study center, the protocol, ICF, patient materials, and other relevant study documents will be submitted to the Ethics Committee for review and written approval. The study center can only commence or release the Investigational Product (IP) to the Investigator upon obtaining the necessary approvals and study information. Any modifications or extensions to the study, such as protocol amendments or changes to the ICF, require obtaining the renewed approval from the Ethics Committee, and the approved documents must be kept in the study files.

The Investigator will promptly report any new information that could impact subject safety or the study's conduct to the Ethics Committee. Additionally, written summaries of the study's progress will be submitted as required. Upon study completion, the Ethics Committee will be notified accordingly.

All protocol amendments will be accurately documented on a protocol amendment form, signed, and dated by the original approving signatories. These amendments will be submitted to the institutional Ethics Committee for approval before implementation, unless there is an immediate hazard to the trial participants, in which case the necessary action will be taken first, followed by the relevant protocol amendment. When protocol amendments or consent form modifications are implemented at the lead site, WCM, updated documents will be shared with participating sites, ensuring that all changes are approved by WCM before local IRB submission.

As per local and national requirements, relevant study documentation will be submitted to the regulatory authorities of the participating countries for review and approval before the study commences. At the conclusion of the study, the regulatory authorities will be notified of its completion.

10.2.2 Ethical Conduct of the Study

The study will be conducted by the Investigators and all involved parties in accordance with ethical principles outlined in the Declaration of Helsinki, Good Clinical Practice (GCP), International Council for Harmonisation (ICH) guidelines, as well as relevant national and local laws and regulatory requirements.

The study will be carried out based on a protocol that has been thoroughly reviewed and approved by the appropriate ethics committees. Investigations will be conducted by qualified individuals with scientific and medical expertise, ensuring that the benefits of the study are balanced with the associated risks.

10.2.3 Informed Consent

The investigator or a qualified representative must obtain documented consent from each potential subject or their legally authorized representative prior to their participation in the research study, following the guidelines of ICH-GCP and applicable local regulations. Subjects who agree to participate will sign the approved ICF and receive a copy of the signed document.

The initial ICF, as well as any subsequent revisions and written information provided to the subjects, must receive approval from the IRB before use. The ICF will comply with IRB/IECrequirements, relevant laws, and regulations.

The informed consent process is ongoing and involves a detailed discussion between study staff and participants via Zoom. Participants will have the opportunity to ask questions and will be given sufficient time to read the consent document and make an informed decision about their participation. Electronic copies of study information and consent forms will be provided to participants for reference, stored on the WCM server.

During the informed consent process, the study personnel will address key points, such as the purpose of the research study, its funding source, and who is responsible for conducting the research. Participants will be informed about the selection process for participation and the potential use of emergency contact information. Detailed explanations will be provided regarding the expectations, time commitment, potential risks and benefits, and any associated costs of participation. Participants will also be made aware that they may experience grief, sadness, or anxiety during their use of the web app. Emphasis will be placed on maintaining participant confidentiality, their rights, and the voluntary nature of their participation, including the right to withdraw at any time.

Caregivers will be asked if the ADRD patient for whom they care would be capable of responding meaningfully to questions about the LMH-4-DCP intervention; we will only approach those patients whom the family caregiver considers willing and able to answer basic demgraphic question on behalf of the PwD.

With participant consent, all interviews will be audio-recorded and transcribed. Participants will still be allowed to take part in the study if they do not give their consent to be recorded. All audio files will be destroyed once transcription is completed. Participants will be interviewed in private settings (whether in-person, via phone, or Zoom) to retrieve information relevant to the aims of the study. Interviews will be scheduled at times convenient for study participants. Each interview will be conducted by a trained interviewer. Content for reaction and a manageable list of questions will be carefully constructed to ease participant burden. Interviews will be asked only essential information needed to meet the study's objectives.

The ICF will include a section explaining protected health information and how it remains confidential according to Health Insurance Portability and Accountability Act (HIPAA) guidelines. Contact information for the Principal Investigators and the Office for the Protection of Research Subjects will be provided in the ICF. All informed consent processes will adhere to the policies established by the Institutional Review Board.

10.2.4 Compliance with Trial Registration and Results Posting Requirements

In accordance with the Food and Drug Administration Modernization Act (FDAMA) and the Food and Drug Administration Amendments Act (FDAAA), the Sponsor-Investigator of the trial holds the responsibility for determining whether the trial and its results need to be submitted to http://www.clinicaltrials.gov. By providing this information, individuals can identify relevant trials for their specific medical conditions and obtain further details on trial locations and contact information by reaching out to a central contact number. This allows them to explore the possibility of participating in suitable trials.

10.2.5 Record Retention

Essential documents are a collection of records that enable the evaluation of the study and the quality of the generated data. Upon completion of the study, the Investigator will maintain all documents and data related to the study in a secure study file, ensuring an organized arrangement. Essential documents should be preserved for a minimum of 2 years after the final marketing approval in an ICH region or for at least 2 years following the cessation of clinical development of the Investigational Product (IP). Furthermore, subject medical records and other source documentation will be retained for the maximum duration allowed by the hospital, institution, or medical practice.

11. Statistical Considerations

Phase 1. Analysis of relevant expert feedback: Relevant expert feedback will be obtained via survey and interviews after the experts have visited the LMH-4-DCP Version 1.0. They will be prompted for feedback on usability, including content and language; design, and aesthetics (colors, feature creep); satisfaction with features/activities; encountered problems; suggestions for website improvement. Interviews will be audio recorded, transcribed and content-analyzed, a well-established, systematic qualitative analysis approach to identify themes in text. We will follow Morse's guidelines for conducting qualitative research (e.g., audit trail, saturation). Transcripts will be reviewed, and key points summarized by study team members with experience conducting qualitative analysis (e.g., Drs. Falzarano, Prigerson). Should we not reach saturation, we will recruit an additional five relevant experts until saturation is achieved. The team will then convene to review relevant expert feedback, generate and discuss a list of proposed modifications, and votes taken to decide on modifications to incorporate into LMH-4-DCP Version 2.0.

Phase 2: Anticipated outcomes of this study include the collection of self-report survey data, behavioral data pertaining to participants' interactions with the LMH-4-DCP web app (such as the duration and activities performed), as well as textual data encompassing participants' interactions with one another within the web app.

Descriptive statistics will characterize recruitment, subjects, reasons for refusal, attrition, and feasibility and acceptability by examining rates of usefulness and satisfaction, login frequency, activities completed and time in each LMH activity. The Actor Partner Interdependence Model (APIM)[54] using structural equation modeling will be used to perform data analysis for caregivers to compare changes in our primary outcomes (pre- loss grief and relationship quality) from baseline to 2-week follow-up in the intervention and control groups. We will compute a ratio of partner and actor effects, which will then be regressed on our two primary outcomes of interest (pre-loss grief and relationship quality). Separate models will be tested for control group and intervention participants for each outcome. Although underpowered, we will explore associations of psychosocial targets significantly related to the predictor and outcome variables, those of which demonstrate significant associations will be examined as potential mediating influences (psychosocial deprivations) in the relationship between LMH-4- DCP use and caregiver pre-loss grief and relationship quality outcomes.

12. References

1. Maciejewski, P. K., Falzarano, F. B., She, W. J., Lichtenthal, W. G., & Prigerson, H. G. (2022). A micro-sociological theory of adjustment to loss. *Current opinion in psychology*, *43*, 96–101. https://doi.org/10.1016/j.copsyc.2021.06.016
2. Alzheimer's Association. (2024). 2024 Alzheimer's disease facts and figures. Alzheimer's & Dementia: The Journal of the Alzheimer's Association, 20(5), 3708-3821. https://doi.org/10.1002/alz.13016
3. Comas-Herrera, A. (2020). Building on hope or tackling fear? Policy responses to the

growing costs of Alzheimer's disease and other dementias. *Wharton Pension Research Council Working Papers*, 688. https://repository.upenn.edu/prc_papers/688

4. Blandin, K., & Pepin, R. (2017). Dementia grief: A theoretical model of a unique grief experience. *Dementia* (London, England), *16*(1), 67-78. https://doi.org/10.1177/1471301215581081
5. Holley, C. K., & Mast, B. T. (2009). The impact of anticipatory grief on caregiver burden in dementia caregivers. *The Gerontologist*, *49*(3), 388-396. https://doi.org/10.1093/geront/gnp061
6. Chan, D., Livingston, G., Jones, L., & Sampson, E. L. (2013). Grief reactions in dementia carers: A systematic review. *International Journal of Geriatric Psychiatry*, *28*(1), 1-17. https://doi.org/10.1002/gps.3795
7. Garand, L., Lingler, J. H., Deardorf, K. E., et al. (2012). Anticipatory grief in new family caregivers of persons with mild cognitive impairment and dementia. *Alzheimer Disease and Associated Disorders*, *26*(2), 159-165. https://doi.org/10.1097/WAD.0b013e31822f9051
8. Wilson, R. S., Boyle, P. A., Segawa, E., et al. (2013). The influence of cognitive decline on well-being in old age. *Psychology and Aging*, *28*(2), 304-313. https://doi.org/10.1037/a0031196
9. Meuser, T. M., & Marwit, S. J. (2001). A comprehensive, stage-sensitive model of grief in dementia caregiving. *The Gerontologist, 41*(5), 658-670. https://doi.org/10.1093/geront/41.5.658
10. Noyes, B. B., Hill, R. D., Hicken, B. L., et al. (2010). The role of grief in dementia caregiving. *American Journal of Alzheimer's Disease & Other Dementias, 25*(1), 9-17. https://doi.org/10.1177/1533317509333902
11. Pearlin, L. I., Mullan, J. T., Semple, S. J., & Skaff, M. M. (1990). Caregiving and the stress process: An overview of concepts and their measures. *The Gerontologist, 30*(5), 583-594. https://doi.org/10.1093/geront/30.5.583
12. Falzarano, F., Prigerson, H. G., & Maciejewski, P. K. (2021). The role of advance care planning in cancer patient and caregiver grief resolution: Helpful or harmful? *Cancers, 13*(8), 1977. https://doi.org/10.3390/cancers13081977
13. Afifi, T. D., Basinger, E. D., & Kam, J. A. (2020). The extended theoretical model of communal coping: Understanding the properties and functionality of communal coping. *Journal of Communication, 70*(3), 424-446. https://doi.org/10.1093/joc/jqaa006
14. Moon, H., Townsend, A. L., Whitlatch, C. J., & Dilworth-Anderson, P. (2017). Quality of life for dementia caregiving dyads: Effects of incongruent perceptions of everyday care and values. *The Gerontologist, 57*(4), 657-666. https://doi.org/10.1093/geront/gnw055
15. Chiambretto, P., Moroni, L., Guarnerio, C., Bertolotti, G., & Prigerson, H. G. (2010). Prolonged grief and depression in caregivers of patients in vegetative state. *Brain Injury, 24*(4), 581-588. https://doi.org/10.3109/02699051003610490
16. Givens, J. L., Prigerson, H. G., Kiely, D. K., Shaffer, M. L., & Mitchell, S. L. (2011). Grief among family members of nursing home residents with advanced dementia. *American Journal of Geriatric Psychiatry, 19*(6), 543-550.

https://doi.org/10.1097/JGP.0b013e31820dcbe0
17. Kiely, D. K., Prigerson, H., & Mitchell, S. L. (2008). Health care proxy grief symptoms before the death of nursing home residents with advanced dementia. *American Journal of Geriatric Psychiatry, 16*(8), 664-673. https://doi.org/10.1097/JGP.0b013e3181784143
18. Schulz, R., Mendelsohn, A. B., Haley, W. E., et al. (2003). End-of-life care and the effects of bereavement on family caregivers of persons with dementia. *New England Journal of Medicine, 349*(20), 1936-1942. https://doi.org/10.1056/NEJMsa035373
19. Simon, N. M. (2013). Treating complicated grief. *JAMA, 310*(4), 416-423. https://doi.org/10.1001/jama.2013.8614
20. Smigelsky, M. A., Bottomley, J. S., Relyea, G., & Neimeyer, R. A. (2020). Investigating risk for grief severity: Attachment to the deceased and relationship quality. *Death Studies, 44*(7), 402-411. https://doi.org/10.1080/07481187.2018.1548539
21. Kokou-Kpolou, C. K., Park, S., Lenferink, L. I. M., Iorfa, S. K., Fernández-Alcántara, M., Derivois, D., & Cénat, J. M. (2021). Prolonged grief and depression: A latent class analysis. *Psychiatry Research*, *299*, 113864. https://doi.org/10.1016/j.psychres.2021.113864
22. Van Doorn, C., Kasl, S. V., Beery, L. C., Jacobs, S. C., & Prigerson, H. G. (1998). The influence of marital quality and attachment styles on traumatic grief and depressive symptoms. *Journal of Nervous and Mental Disease, 186*(9), 566-573. https://doi.org/10.1097/00005053-199809000-00008
23. Holland, J. M., Currier, J. M., & Gallagher-Thompson, D. (2009). Outcomes from the Resources for Enhancing Alzheimer's Caregiver Health (REACH) program for bereaved caregivers. *Psychology and Aging, 24*(1), 190-202. https://doi.org/10.1037/a0014303
24. Jacobsen, J. C., Zhang, B., Block, S. D., Maciejewski, P. K., & Prigerson, H. G. (2010). Distinguishing symptoms of grief and depression in a cohort of advanced cancer patients. *Death Studies, 34*(3), 257-273. https://doi.org/10.1080/07481180903559303
25. Breen, L. J., Aoun, S. M., O'Connor, M., Johnson, A. R., & Howting, D. (2020). Effect of caregiving at end of life on grief, quality of life and general health: A prospective, longitudinal, comparative study. *Palliative Medicine, 34*(1), 145-154. https://doi.org/10.1177/0269216319880766
26. Prigerson, H. G., Viola, M., Brewin, C. R., et al. (2019). Enhancing & Mobilizing the POtential for Wellness & Emotional Resilience (EMPOWER) among surrogate decision-makers of ICU patients: Study protocol for a randomized controlled trial. *Trials, 20*(1), 408. https://doi.org/10.1186/s13063-019-3515-0
27. Maciejewski, P. K., & Prigerson, H. G. (2013). Emotional numbness modifies the effect of end-of-life discussions on end-of-life care. *Journal of Pain and Symptom Management, 45*(5), 841-847. https://doi.org/10.1016/j.jpainsymman.2012.04.003
28. Lichtenthal, W. G., Nilsson, M., Kissane, D. W., et al. (2011). Underutilization of mental health services among bereaved caregivers with prolonged grief disorder. *Psychiatric Services, 62*(10), 1225-1229. https://doi.org/10.1176/ps.62.10.pss6210_1225
29. Prigerson, H. G., Horowitz, M. J., Jacobs, S. C., et al. (2009). Prolonged grief disorder: Psychometric validation of criteria proposed for DSM-V and ICD-11. *PLoS Medicine, 6*(8), e1000121. https://doi.org/10.1371/journal.pmed.1000121

30. Tomarken, A., Holland, J., Schachter, S., et al. (2008). Factors of complicated grief pre-death in caregivers of cancer patients. *Psycho-Oncology, 17*(2), 105-111. https://doi.org/10.1002/pon.1188
31. Maciejewski, P. K., Falzarano, F. B., She, W. J., Lichtenthal, W. G., & Prigerson, H. G. (2022). A micro-sociological theory of adjustment to loss. *Current Opinion in Psychology, 43*, 96-101. https://doi.org/10.1016/j.copsyc.2021.06.016
32. Pfeiffer, E. (1975). A short portable mental status questionnaire for the assessment of organic brain deficit in elderly patients. *Journal of the American Geriatrics Society, 23*(10), 433-441. https://doi.org/10.1111/j.1532-5415.1975.tb00927.x
33. Jeste, D. V., Palmer, B. W., Appelbaum, P. S., et al. (2007). A new brief instrument for assessing decisional capacity for clinical research. *Archives of General Psychiatry, 64*(8), 966–974. https://doi.org/10.1001/archpsyc.64.8.966
34. Seaman, J. B., Terhorst, L., Gentry, A., Hunsaker, A., Parker, L. S., & Lingler, J. H. (2015). Psychometric properties of a decisional capacity screening tool for individuals contemplating participation in Alzheimer's disease research. *Journal of Alzheimer's Disease, 46*(1), 1-9. https://doi.org/10.3233/JAD-142559
35. Song, M. K., Ward, S. E., Hepburn, K., et al. (2019). Can persons with dementia meaningfully participate in advance care planning discussions? A mixed-methods study of SPIRIT. *Journal of Palliative Medicine, 22*(11), 1410-1416. https://doi.org/10.1089/jpm.2019.0088
36. Boot, W. R., Charness, N., Czaja, S. J., Sharit, J., Rogers, W. A., Fisk, A. D., Mitzner, T., Lee, C. C., & Nair, S. (2015). Computer proficiency questionnaire: Assessing low and high computer proficient seniors. *The Gerontologist*, *55*(3), 404–411. https://doi.org/10.1093/geront/gnt117
37. Rosenthal, D. A., Gurney, R. M., & Moore, S. M. (1981). From trust on intimacy: A new inventory for examining Erikson's stages of psychosocial development. *Journal of Youth and Adolescence*, *10*(6), 525-537. https://doi.org/10.1007/BF02087944
38. Van Orden, K. A., Cukrowicz, K. C., Witte, T. K., & Joiner, T. E., Jr. (2012). Thwarted belongingness and perceived burdensomeness: Construct validity and psychometric properties of the Interpersonal Needs Questionnaire. *Psychological Assessment*, *24*(1), 197–215. https://doi.org/10.1037/a0025358
39. Cohen, S., & Hoberman, H. (1983). Positive events and social supports as buffers of life change stress. Journal of Applied Social Psychology, 13, 99-125. https://doi.org/10.1111/j.1559-1816.1983.tb02325.x
40. Pearlin, L. I., Mullan, J. T., Semple, S. J., & Skaff, M. M. (1990). Caregiving and the stress process: An overview of concepts and their measures. *The Gerontologist*, *30*(5), 583-594. https://doi.org/10.1093/geront/30.5.583
41. Tarlow, B. J., Wisniewski, S. R., Belle, S. H., Rubert, M., Ory, M. G., & Gallagher-Thompson, D. (2004). Positive aspects of caregiving: Contributions of the REACH project to the development of new measures for Alzheimer's caregiving. *Research on Aging*, *26*(4), 429-453. https://10.1177/0164027504264493
42. Lubben, J., Blozik, E., Gillmann, G., Iliffe, S., von Renteln Kruse, W., Beck, J. C., & Stuck, A.

E. (2006). Performance of an abbreviated version of the Lubben Social Network Scale among three European community-dwelling older adult populations. The Gerontologist, 46(4), 503-513. https://doi.org/10.1093/geront/46.4.503

43. Kroenke, K., Spitzer, R. L., & Williams, J. B. (2001). The PHQ-9: validity of a brief depression severity measure. *Journal of General Internal Medicine*, *16*(9), 606–613. https://doi.org/10.1046/j.1525-1497.2001.016009606.x
44. Bédard, M., Molloy, D. W., Squire, L., Dubois, S., Lever, J. A., & O'Donnell, M. (2001). The Zarit Burden Interview: a new short version and screening version. *The Gerontologist*, *41*(5), 652–657. https://doi.org/10.1093/geront/41.5.652
45. Spitzer, R. L., Kroenke, K., Williams, J. B., & Löwe, B. (2006). A brief measure for assessing generalized anxiety disorder: the GAD-7. *Archives of internal medicine*, *166*(10), 1092–1097. https://doi.org/10.1001/archinte.166.10.1092
46. The WHOQOL Group. (1998). Development of the World Health Organization WHOQOL-BREF quality of life assessment. Psychological Medicine, 28(3), 551–558. https://doi.org/10.1017/s0033291798006667
47. Brooke, J. (1986). SUS: A quick and dirty usability scale. Digital Equipment Corporation.
48. Lewis, J. R. (2018). The system usability scale: past, present, and future. *International Journal of Human–Computer Interaction*, *34*(7), 577-590. https://doi.org/10.1080/10447318.2018.1455307
49. Givens, J.L., Prigerson, H.G., Kiely, D.K., Shaffer, M.L., Mitchell, S.L. (2011). Grief among family members of nursing home residents with advanced dementia. Am. J. Geriatr. Psychiatry, 19, 543–550. https://doi.org/10.1097/JGP.0b013e31820dcbe0
50. Jacobsen, J.C., Zhang, B., Block, S.D., Maciejewski, P.K., & Prigerson, H.G. (2010). Distinguishing symptoms of grief and depression in a cohort of advanced cancer patients. Death Studies, 34, 257–273. https://doi.org/10.1080/07481180903559303
51. Pearlin, L. I., Mullan, J. T., Semple, S. J., & Skaff, M. M. (1990). Caregiving and the stress process: An overview of concepts and their measures. *The Gerontologist*, *30*(5), 583-594. https://doi.org/10.1093/geront/30.5.583
52. Reid, C. E., Moss, S., & Hyman, G. (2005). Caregiver reciprocity: The effect of reciprocity, carer self-esteem and motivation on the experience of caregiver burden. *Australian Journal of Psychology*, *57*(3), 186-196. https://doi.org/10.1080/00049530500141022
53. Williamson, G. M., & Shaffer, D. R. (2001). Relationship quality and potentially harmful behaviors by spousal caregivers: how we were then, how we are now. The Family Relationships in Late Life Project. *Psychology and Aging*, *16*(2), 217–226. https://doi.org/10.1037/08827974.16.2.217
54. Cook, W. L., & Kenny, D. A. (2005). The actor–partner interdependence model: A model of bidirectional effects in developmental studies. *International Journal of Behavioral Development, 29*(2), 101-109. https://doi.org/10.1080/01650250444000405
