# Supplementary material for: Digital Reminiscence for Predeath Grief Among Family Caregivers of Patients With Dementia: A Pilot Randomized Clinical Trial
Source: JAMA Netw Open. 2026 Apr 22;9(4):e268278. doi: 10.1001/jamanetworkopen.2026.8278 (PMC13103805; doi:10.1001/jamanetworkopen.2026.8278)
Supplement: Supplement 2. — eAppendix. Acceptability Questionnaire Item-Level Responses [file jamanetwopen-e268278-s002.pdf]

Supplementary Online Content

Falzarano FB, Greenfield A, Saviano SC, et al. Digital reminiscence for predeath grief among family caregivers of patients with dementia: a pilot randomized clinical trial. *JAMA Netw Open.* 2026;9(4):e268278. doi:10.1001/jamanetworkopen.2026.8278

eAppendix. Acceptability Questionnaire Item-Level Responses

This supplementary material has been provided by the authors to give readers additional information about their work.

© 2026 Falzarano FB et al. *JAMA Network Open*.

Item	n (%)
1. I found the LMH-4-DCP application easy to use.	
Strongly Disagree	0
Disagree	0
Neither Agree nor Disagree	4 (14.8)
Agree	13 (48.1)
Strongly Agree	10 (37.0)
2.I enjoyed my experience using the LMH-4-DCP application.	
Strongly Disagree	0
Disagree	1 (3.7)
Neither Agree nor Disagree	5 (18.5)
Agree	10 (37.0)
Strongly Agree	11 (40.7)
3. I found the application's tutorials and instructions easy to follow.	
Strongly Disagree	1 (3.7)
Disagree	0
Neither Agree nor Disagree	2 (7.4)
Agree	13 (48.1)
Strongly Agree	11 (40.7)
4. Using the LMH-4-DCP application was worth my time.	
Strongly Disagree	1 (3.7)
Disagree	1 (3.7)
Neither Agree nor Disagree	3 (11.1)
Agree	10 (37.0)
Strongly Agree	12 (44.4)
5. I was satisfied with the features in the Writing Room.	
Strongly Disagree	1 (3.7)
Disagree	1 (3.7)
Neither Agree nor Disagree	5 (18.5)
Agree	12 (44.4)
Strongly Agree	8 (29.6)
6. I was satisfied with the features in the Reminiscence Room.	
Strongly Disagree	1 (3.7)
Disagree	0
Neither Agree nor Disagree	1 (3.7)
Agree	15 (55.6)
Strongly Agree	10 (37.0)
7. I was satisfied with the features in the Reading Room.	
Strongly Disagree	2 (7.4)
Disagree	0
Neither Agree nor Disagree	3 (11.1)
Agree	14 (51.9)
Strongly Agree	8 (29.6)

© 2026 Falzarano FB et al. *JAMA Network Open*.

8. I would like to continue to use the LMH-4-DCP application after my participation in this study.	
Strongly Disagree	0
Disagree	3 (11.1)
Neither Agree nor Disagree	3 (11.1)
Agree	12 (44.4)
Strongly Agree	19 (33.3)

Note. LMH-4-DCP = Living Memory Home for Dementia Care Pairs; Intervention participants only n=27.

© 2026 Falzarano FB et al. *JAMA Network Open*.
