# Supplementary material for: Digital Reminiscence for Predeath Grief Among Family Caregivers of Patients With Dementia: A Pilot Randomized Clinical Trial
Source: JAMA Netw Open. 2026 Apr 22;9(4):e268278. doi: 10.1001/jamanetworkopen.2026.8278 (PMC13103805; doi:10.1001/jamanetworkopen.2026.8278)
Supplement: Supplement 3. — Data Sharing Statement [file jamanetwopen-e268278-s003.pdf]

Data Sharing Statement

Falzarano. Digital Reminiscence for Predeath Grief Among Family Caregivers of Patients With Dementia. *JAMA Netw Open*. Published April 22, 2026.
doi:10.1001/jamanetworkopen.2026.8278

Data
Additional Information: Trial Registration: Clinicaltrials.gov: NCT06225986, Registered: January 9, 2024, ClinicalTrials.gov; https://clinicaltrials.gov/study/NCT06225986?term=living%20memory%20home&rank=1
Data available: Yes
Data types: Deidentified participant data, Data dictionary
How to access data: Data will be available upon reasonable request to the study's corresponding author at falzaran@usc.edu.
When available: With publication

Supporting Documents
Document types: None

Additional Information
Who can access the data: Data will be made available to researchers whose proposed use of the data has been approved by the study's Multiple Principal Investigators (MPIs: Falzarano, Prigerson).
Types of analyses: For any purpose.
Mechanisms of data availability: Data will be made available upon approval of a proposal by the study's Multiple Principal Investigator (MPI) team and the execution of relevant signed data access agreements.
